# Data on a genome-wide association study of type 2 diabetes in a Maya population

**DOI:** 10.1016/j.dib.2019.104866

**Published:** 2019-11-22

**Authors:** Armando Totomoch-Serra, Miriam Givisay Domínguez-Cruz, María de Lourdes Muñoz, María Guadalupe García-Escalante, Juan Burgueño, Álvaro Díaz-Badillo, Nina Valadez-González, Doris Pinto Escalante

**Affiliations:** a Department of Genetics and Molecular Biology, Centro de Investigación y de Estudios Avanzados Del Instituto Politécnico Nacional, Mexico City, Mexico; b PhD Program in Medical Sciences, Universidad de La Frontera, Chile; c Laboratorios de Genética y Hematología, Centro de Investigaciones Regionales “Dr. Hideyo Noguchi”, Universidad Autónoma de Yucatán, Mérida, Yucatán, Mexico; d Centro Internacional de Mejoramiento de Maíz y Trigo. El Batán, Texcoco, State of Mexico, Mexico; e Maestría en Salud Publica, Universidad México Americana Del Norte, Reynosa, Tamaulipas, Mexico; f Department of Epidemiology, Human Genetics & Environmental Sciences, The University of Texas Health Science Center at Houston, Brownsville, TX, USA

**Keywords:** Microarray, Native mexicans, Genetic markers, Ancestry, Replication of T2D variants

## Abstract

Maya communities have been shown to exhibit type 2 diabetes (T2D) with high prevalence compared with Mexican mestizo populations. Furthermore, some variants associated with the risk for T2D have been described. In this study, we describe the results of a pilot genome wide association study (GWAS) using 817,823 single nucleotide polymorphisms (SNPs) to identify candidate variants for replication in future studies. Herein, we present the GWAS study data, which were divided into three parts: first, 1289 ancestry informative markers (AIMs) were selected for Latino populations containing European, African, and Native American SNPs obtained from the literature; second, a GWAS hypothesis free to select candidate genes associated with T2D was performed, which identified 24 candidate genes; and third, 39 SNPs previously associated with T2D or related traits were replicated. This article is associated with the original article published in “Gene” under the title **“**Pilot genome-wide association study identifying novel risk loci for type 2 diabetes in a Maya population”.

**Table:** Specifications Table

Subject area	Genetics, Genomics and Molecular Biology.
More specific subject area	Diabetes and Maya populations
Type of data	Tables, figures and text file
How data was acquired	All the genotypic data was obtained by “Centro de Investigaciones y Estudios Avanzados del Instituto Politécnico Nacional (CINVESTAV)” by analysis of the DNA obtained from blood samples of volunteer donors acquired from the primary health centre located in a rural and an urban community in the State of Yucatán, México by the “Centro de Investigaciones Regionales Dr. Hideyo Noguchi, Universidad Autónoma de Yucatán”. The genotypic data obtained from the microarray “Affymetrix Axiom Genome-wide LAT1 array” were analysed by CINVESTAV and “Centro Internacional de Mejoramiento de Maíz y Trigo”.
Data format	Original and analysed dataset.
Experimental factors	Patients were recruited in 2010, and DNA was extracted from whole blood using the automated extraction system Chemagic Prepito® (PerkinElmer Inc., Waltham, MA, USA) according to the manufacturer's recommendations.
Experimental features	Affymetrix Axiom Genome-wide LAT1 array.
Data source location	Centro de Investigaciones y Estudios Avanzados del Instituto Politécnico Nacional, Mexico City, Mexico.
Data accessibility	Data are available with this article.
Related research article	Domínguez-Cruz MG, Muñoz ML, Totomoch-Serra A, García-Escalante MG, Burgueño J, Valadez-González N, Pinto-Escalantes D, Díaz-Badillo. A pilot genome-wide association study identifying novel risk loci for type 2 diabetes in a Maya population. Pilot genome-wide association study identifying novel risk loci for type 2 diabetes in a Maya population. Gene, 2018, Nov. 30; 677:324–331. https://doi.org/10.1016/j.gene.2018.08.041. Epub 2018 Aug 18.

**Table:** 

**Value of the Data** • New genotypic data in a Maya population with T2D compared with a non-diabetic Maya population, which is scarce in the literature. • These data contain the descriptive data of 24 candidate variants associated with T2D in a Maya population. • Complementary data for the *AGTR2* rs1914711 associated with T2D in a Maya population • The results obtained could also be used for comparison in future genetic association studies in Mexican patients with T2D. • Ancestry markers are included.

## 1 Data

A pilot genome-wide association study was performed in a Maya population with high prevalence of T2D and compared with a group of healthy Maya volunteers using statistical models for genotype, allelic, and Armitage trend tests [1].

The structure of the Maya population was determined by calculating the allelic frequencies of 1289 ancestry markers for a Latino population containing European, African, and Native American SNPs described previously in the literature (Table 1). The most significant informative markers for the Maya population were localized on chromosomes 22, 19 and 15. In addition, 24 variants located in 15 chromosomes were proposed as candidates for future replication in different populations (Table 2). The *AGTR2* rs1914711 variant (OR = 6.824; p = 1.448 × 10 -9) located on chromosome X was associated with the risk of T2D by the allele test, and the *ARL15* rs4311394 variant (OR = 0.318; p = 0.001) was associated with protection against T2D by the genotype and the Armitage trend test.

**Table 1:** Description of ancestry markers selected from the literature.

Chr	SNP-Array ID	dbSNP	Type	Pos	AF G1	AF G2	Global Frequency	Frequency Group	Group
1	snp_rs241257_0w_f	rs241257	AncAm	4614822	0.498	0.805	0.30678	4	G2
1	hmrs2012852_r	rs2012852	AIMS	5541205	0.244	0.361	0.11754	2	G2
1	hmrs4908736_f	rs4908736	AIMS	8187211	0.547	0.259	0.28788	3	G1
1	snp_rs703804_0w_f	rs703804	AIMS	12868237	0.578	0.790	0.21121	3	G2
1	snp_rs7414562_1wa0_f	rs7414562	AncAm	14359780	0.383	0.018	0.36501	4	G1
1	snp_rs11249024_0w_f	rs11249024	AncAm	24527095	0.669	0.238	0.43155	5	G1
1	snp_rs1981135_0w_r	rs1981135	AIMS	26447739	0.042	0.000	0.04207	1	G1
1	hmrs669538_f	rs669538	AIMS	32711360	0.474	0.615	0.14092	2	G2
1	snp_rs6665029_0w_f	rs6665029	AIMS	35040991	0.111	0.349	0.23821	3	G2
1	snp_rs1334804_0w_f	rs1334804	AIMS	37395244	0.042	0.000	0.04195	1	G1
1	snp_rs849961_0w_f	rs849961	AncAm	37577214	0.552	0.839	0.28706	3	G2
1	snp_rs898979_0w_f	rs898979	AIMS	38666914	0.071	0.177	0.10542	2	G2
1	snp_rs10889750_0w_f	rs10889750	AIMS	41411249	0.612	0.866	0.25436	3	G2
1	snp_rs11581921_0w_f	rs11581921	AIMS	43215930	0.042	0.054	0.01194	1	G2
1	hmrs4233497_f	rs4233497	AIMS	45763753	0.374	0.054	0.32035	4	G1
1	snp_rs4660344_0w_r	rs4660344	AIMS	46805222	0.496	0.409	0.08760	1	G1
1	snp_rs12075035_0w_35CT_r	rs12075035	AIMS	52023412	0.243	0.228	0.01534	1	G1
1	snp_rs1288367_0w_f	rs1288367	AIMS	53605884	0.058	0.105	0.04748	1	G2
1	snp_rs12130799_0w_f	rs12130799	AIMS	55663372	0.192	0.349	0.15730	2	G2
1	snp_rs17469007_0w_f	rs17469007	AIMS	56872794	0.301	0.333	0.03270	1	G2
1	snp_rs12407896_0w_r	rs12407896	AncAm	57933955	0.321	0.037	0.28335	3	G1
1	snp_rs973236_0w_f	rs973236	AIMS	58769757	0.042	0.000	0.04219	1	G1
1	snp_rs10889092_0w_r	rs10889092	AncAm	58915024	0.399	0.074	0.32529	4	G1
1	snp_rs2984915_0w_r	rs2984915	AIMS	59253695	0.075	0.033	0.04217	1	G1
1	snp_rs3767996_0w_r	rs3767996	AIMS	60281712	0.263	0.393	0.13010	2	G2
1	snp_rs4915746_0w_r	rs4915746	AncAm	61952574	0.516	0.214	0.30246	4	G1
1	hmrs11207865_f	rs11207865	AIMS	62387652	0.416	0.152	0.26363	3	G1
1	snp_rs6681578_0w_f	rs6681578	AIMS	65013586	0.314	0.000	0.31384	4	G1
1	snp_rs310199_0w_r	rs310199	AncAm	65350122	0.450	0.851	0.40105	5	G2
1	snp_rs1566246_0w_r	rs1566246	AIMS	67245275	0.363	0.275	0.08800	1	G1
1	snp_rs357234_0w_f	rs357234	AncAm	72015414	0.609	0.067	0.54228	6	G1
1	snp_rs3101336_0w_f	rs3101336	AIMS	72751185	0.688	0.789	0.10069	2	G2
1	snp_rs1360244_0w_r	rs1360244	AIMS	74058924	0.123	0.366	0.24303	3	G2
1	snp_rs544858_0w_f	rs544858	AIMS	76725259	0.307	0.187	0.11968	2	G1
1	snp_rs12022561_0w_r	rs12022561	Both	80235819	0.430	0.765	0.33421	4	G2
1	snp_rs12758138_0w_f	rs12758138	AIMS	81230013	0.685	0.655	0.03063	1	G1
1	snp_rs962249_0w_f	rs962249	AIMS	82561559	0.581	0.352	0.22890	3	G1
1	hmrs11163752_r	rs11163752	AIMS	83934440	0.243	0.511	0.26775	3	G2
1	snp_rs10923099_1wa0_f	rs10923099	AIMS	89009265	0.101	0.134	0.03335	1	G2
1	snp_rs6660484_0w_f	rs6660484	AIMS	92172608	0.423	0.273	0.14957	2	G1
1	snp_rs497511_0w_f	rs497511	AIMS	94523113	0.403	0.880	0.47698	5	G2
1	snp_rs2968485_0w_f	rs2968485	AIMS	96869933	0.204	0.662	0.45792	5	G2
1	snp_rs712886_0w_f	rs712886	AIMS	99751518	0.164	0.165	0.00074	1	G2
1	snp_rs12061503_0w_r	rs12061503	AIMS	101843254	0.150	0.091	0.05856	1	G1
1	snp_rs12046602_0w_f	rs12046602	AIMS	105659063	0.140	0.017	0.12290	2	G1
1	snp_rs877068_0w_r	rs877068	AncAm	110698045	0.284	0.078	0.20625	3	G1
1	snp_rs11485101_0w_f	rs11485101	AIMS	114501968	0.110	0.037	0.07302	1	G1
1	snp_rs544173_0w_f	rs544173	AIMS	118300750	0.441	0.555	0.11337	2	G2
1	snp_rs10923574_0w_r	rs10923574	AncAm	119030698	0.404	0.914	0.51022	6	G2
1	snp_rs10923724_0w_r	rs10923724	AIMS	119546842	0.278	0.419	0.14099	2	G2
1	snp_rs1298954_0w_f	rs1298954	AIMS	145730160	0.342	0.432	0.08981	1	G2
1	snp_rs6587597_0w_f	rs6587597	AIMS	151520731	0.756	0.133	0.62353	7	G1
1	snp_rs12754243_0w_f	rs12754243	AIMS	152685025	0.180	0.029	0.15121	2	G1
1	snp_rs1778826_0w_f	rs1778826	AIMS	156538730	0.286	0.467	0.18101	2	G2
1	snp_rs2814778_0w_f	rs2814778	AIMS	159174683	0.067	0.028	0.03904	1	G1
1	snp_rs4656236_1wa0_r	rs4656236	AIMS	159410975	0.761	0.956	0.19479	2	G2
1	snp_rs11265582_0w_r	rs11265582	AncAm	161255333	0.777	0.167	0.61024	7	G1
1	snp_rs1419074_0w_f	rs1419074	AIMS	165020001	0.139	0.035	0.10367	2	G1
1	snp_rs4657449_0w_f	rs4657449	AncAm	165465281	0.529	0.330	0.19907	2	G1
1	snp_rs10753757_0w_f	rs10753757	AIMS	167382374	0.200	0.022	0.17786	2	G1
1	snp_rs10918767_0w_f	rs10918767	AncAm	167763804	0.439	0.746	0.30657	4	G2
1	snp_rs1412337_0w_f	rs1412337	AIMS	168618641	0.766	0.888	0.12138	2	G2
1	snp_rs12406353_0w_f	rs12406353	AIMS	171972317	0.085	0.133	0.04749	1	G2
1	snp_rs859362_0w_f	rs859362	AIMS	175495090	0.891	0.821	0.06980	1	G1
1	snp_rs6680701_0w_r	rs6680701	AIMS	177313278	0.576	0.578	0.00165	1	G2
1	snp_rs10753193_0w_f	rs10753193	AIMS	179334984	0.073	0.078	0.00515	1	G2
1	snp_rs558994_0w_f	rs558994	AIMS	181477525	0.566	0.803	0.23679	3	G2
1	snp_rs6424913_0w_r	rs6424913	AIMS	183798877	0.357	0.679	0.32129	4	G2
1	snp_rs2146060_0w_f	rs2146060	AIMS	186243582	0.084	0.000	0.08405	1	G1
1	snp_rs4255388_0w_r	rs4255388	AIMS	188770589	0.426	0.262	0.16451	2	G1
1	snp_rs2419063_0w_f	rs2419063	AIMS	190256835	0.096	0.496	0.39933	4	G2
1	hmrs10921094_r	rs10921094	AIMS	192114806	0.026	0.010	0.01537	1	G1
1	snp_rs1339951_0w_f	rs1339951	AIMS	196482357	0.098	0.071	0.02630	1	G1
1	snp_rs4639796_0w_r	rs4639796	AncAm	197126649	0.201	0.659	0.45875	5	G2
1	snp_rs2784101_0w_r	rs2784101	AIMS	198757361	0.731	0.646	0.08431	1	G1
1	hmrs2204309_r	rs2204309	AIMS	200199971	0.452	0.388	0.06434	1	G1
1	snp_rs1400875_0w_r	rs1400875	AIMS	201821443	0.250	0.372	0.12270	2	G2
1	snp_rs2494287_0w_f	rs2494287	AncAm	203207520	0.243	0.622	0.37898	4	G2
1	snp_rs4844484_0w_r	rs4844484	AIMS	209837683	0.621	0.895	0.27401	3	G2
1	snp_rs4532912_0w_r	rs4532912	AIMS	211021787	0.685	0.578	0.10709	2	G1
1	snp_rs351408_0w_r	rs351408	AIMS	212441128	0.330	0.315	0.01562	1	G1
1	snp_rs2818964_0w_r	rs2818964	AIMS	216682448	0.367	0.256	0.11083	2	G1
1	snp_rs10779334_0w_r	rs10779334	AIMS	219106404	0.104	0.087	0.01687	1	G1
1	snp_rs4373767_0w_r	rs4373767	AIMS	219759682	0.612	0.598	0.01441	1	G1
1	snp_rs2889895_0w_f	rs2889895	AIMS	220828130	0.439	0.147	0.29187	3	G1
1	snp_rs17163128_0w_r	rs17163128	AncAm	222619902	0.151	1.000	0.84895	9	G2
1	snp_rs7517868_0w_r	rs7517868	AIMS	224251666	0.498	0.190	0.30767	4	G1
1	hmrs7524430_f	rs7524430	AIMS	226693542	0.045	0.034	0.01082	1	G1
1	snp_rs1930903_0w_r	rs1930903	AIMS	229861486	0.489	0.320	0.16838	2	G1
1	snp_rs2275302_0w_r	rs2275302	AIMS	232590866	0.317	0.929	0.61144	7	G2
1	snp_rs7546393_0w_f	rs7546393	AIMS	233597351	0.000	0.062	0.06231	1	G2
1	snp_rs270505_0w_f	rs270505	AIMS	234600919	0.458	0.322	0.13592	2	G1
1	snp_rs10802626_0w_f	rs10802626	AIMS	237796837	0.433	0.400	0.03285	1	G1
1	snp_rs3748540_0w_r	rs3748540	AIMS	240693711	0.373	0.108	0.26577	3	G1
1	snp_rs7525142_0w_f	rs7525142	AIMS	240742905	0.200	0.237	0.03692	1	G2
1	snp_rs2490400_0w_f	rs2490400	AIMS	243492643	0.325	0.516	0.19041	2	G2
1	snp_rs9725312_0w_f	rs9725312	AIMS	245038818	0.000	0.053	0.05349	1	G2
1	snp_rs10924125_0w_r	rs10924125	AIMS	245388149	0.099	0.374	0.27561	3	G2
1	snp_rs4916113_0w_f	rs4916113	AIMS	248377292	0.084	0.178	0.09458	1	G2
2	snp_rs2724863_0w_f	rs2724863	AncAm	477364	0.388	0.875	0.48742	5	G2
2	snp_rs4246561_0w_f	rs4246561	AIMS	1152434	0.110	0.081	0.02843	1	G1
2	snp_rs587913_0w_f	rs587913	AIMS	4476883	0.360	0.074	0.28562	3	G1
2	snp_rs10495532_0w_r	rs10495532	AIMS	6084916	0.070	0.000	0.07045	1	G1
2	snp_rs6431897_0w_f	rs6431897	AncAm	7933809	0.668	0.137	0.53106	6	G1
2	snp_rs798443_0w_r	rs798443	AIMS	7968275	0.202	0.379	0.17719	2	G2
2	snp_rs409169_0w_r	rs409169	AIMS	8556084	0.118	0.023	0.09523	1	G1
2	snp_rs2140701_0w_r	rs2140701	AIMS	9807702	0.363	0.047	0.31606	4	G1
2	snp_rs6432155_0w_f	rs6432155	AncAm	11103159	0.299	0.129	0.16990	2	G1
2	hmrs4260216_r	rs4260216	AIMS	11535523	0.315	0.501	0.18624	2	G2
2	snp_rs779914_0w_f	rs779914	AIMS	12700740	0.190	0.235	0.04514	1	G2
2	snp_rs4233905_0w_f	rs4233905	AIMS	13848939	0.211	0.591	0.38015	4	G2
2	snp_rs2193908_0w_f	rs2193908	AIMS	15282571	0.127	0.461	0.33458	4	G2
2	snp_rs956968_0w_r	rs956968	AncAm	16138879	0.619	0.177	0.44151	5	G1
2	snp_rs340747_0w_f	rs340747	AIMS	16452715	0.029	0.372	0.34317	4	G2
2	snp_rs1367158_0w_r	rs1367158	AncAm	16590965	0.573	0.121	0.45205	5	G1
2	snp_rs798364_0w_f	rs798364	AIMS	16899190	0.094	0.100	0.00654	1	G2
2	snp_rs10168917_0w_f	rs10168917	AncAm	18336233	0.502	0.922	0.41943	5	G2
2	snp_rs1489688_0w_f	rs1489688	AIMS	18662253	0.011	0.011	0.00015	1	G1
2	snp_rs1427547_0w_r	rs1427547	AIMS	19739976	0.262	1.000	0.73844	8	G2
2	snp_rs2082871_0w_f	rs2082871	AncAm	20712300	0.490	0.162	0.32734	4	G1
2	snp_rs4665797_0w_r	rs4665797	AIMS	21669776	0.151	0.064	0.08691	1	G1
2	snp_rs1438131_0w_f	rs1438131	AIMS	23506782	0.337	0.222	0.11575	2	G1
2	snp_rs12233132_0w_r	rs12233132	AncAm	25328703	0.377	0.143	0.23359	3	G1
2	snp_rs13032262_0w_r	rs13032262	AIMS	25646321	0.098	0.000	0.09779	1	G1
2	snp_rs6731972_0w_f	rs6731972	AncAm	26151206	0.490	0.312	0.17837	2	G1
2	snp_rs4450561_0w_r	rs4450561	AIMS	26653457	0.662	0.840	0.17817	2	G2
2	snp_rs4666200_0w_f	rs4666200	AIMS	29538411	0.361	0.124	0.23706	3	G1
2	snp_rs605832_0w_f	rs605832	AIMS	31454545	0.551	0.214	0.33733	4	G1
2	snp_rs17338665_0w_f	rs17338665	AIMS	33947012	0.184	0.000	0.18407	2	G1
2	snp_rs10175357_0w_r	rs10175357	AIMS	35161672	0.054	0.054	0.00028	1	G2
2	snp_rs12987048_0w_f	rs12987048	AIMS	37024713	0.690	1.000	0.31008	4	G2
2	snp_rs6734118_0w_r	rs6734118	AncAm	37559355	0.677	0.042	0.63473	7	G1
2	snp_rs4670838_0w_f	rs4670838	AncAm	38482924	0.000	0.663	0.66317	7	G2
2	snp_rs2168043_0w_r	rs2168043	AIMS	39332093	0.337	0.037	0.30013	4	G1
2	snp_rs11124754_0w_f	rs11124754	AncAm	40876113	0.295	0.867	0.57212	6	G2
2	snp_rs7580660_0w_f	rs7580660	AIMS	42136788	0.184	0.557	0.37314	4	G2
2	snp_rs6544718_0w_r	rs6544718	AIMS	44104925	0.124	0.137	0.01220	1	G2
2	snp_rs1124787_0w_r	rs1124787	AIMS	46314935	0.436	0.149	0.28775	3	G1
2	snp_rs6713506_0w_r	rs6713506	AIMS	48038331	0.112	0.000	0.11236	2	G1
2	snp_rs12621581_0w_f	rs12621581	AIMS	50190353	0.678	0.493	0.18574	2	G1
2	snp_rs7577894_0w_f	rs7577894	AIMS	56008904	0.316	0.324	0.00778	1	G2
2	snp_rs10490113_0w_f	rs10490113	AIMS	59499347	0.000	0.312	0.31172	4	G2
2	snp_rs4643526_0w_r	rs4643526	AIMS	61184651	0.782	0.952	0.16948	2	G2
2	snp_rs2075375_0w_r	rs2075375	AIMS	63280690	0.461	0.339	0.12181	2	G1
2	snp_rs1861407_0w_f	rs1861407	AIMS	64939389	0.025	0.082	0.05630	1	G2
2	snp_rs11675929_0w_f	rs11675929	AIMS	66297608	0.287	0.289	0.00205	1	G2
2	snp_rs6715551_0w_f	rs6715551	AIMS	68035962	0.614	0.741	0.12705	2	G2
2	snp_rs7595509_0w_f	rs7595509	AIMS	68116027	0.681	0.184	0.49734	5	G1
2	snp_rs13416937_0w_f	rs13416937	AIMS	74225434	0.895	0.809	0.08578	1	G1
2	snp_rs13032535_0w_f	rs13032535	AIMS	75875317	0.606	0.673	0.06716	1	G2
2	snp_rs7561233_0w_f	rs7561233	AIMS	77029323	0.405	0.340	0.06520	1	G1
2	hmrs1370637_f	rs1370637	AIMS	78341245	0.301	0.431	0.12969	2	G2
2	snp_rs6740609_0w_f	rs6740609	AncAm	79855622	0.367	0.844	0.47702	5	G2
2	snp_rs6742029_0w_r	rs6742029	AIMS	80716691	0.430	0.429	0.00107	1	G1
2	snp_rs4144835_0w_r	rs4144835	AncAm	84309778	0.656	0.212	0.44452	5	G1
2	snp_rs1358192_0w_r	rs1358192	AIMS	84371635	0.528	0.544	0.01586	1	G2
2	snp_rs11682587_0w_f	rs11682587	AIMS	86677496	0.019	0.023	0.00403	1	G2
2	snp_rs17435433_0w_f	rs17435433	AncAm	88509616	0.547	0.919	0.37154	4	G2
2	snp_rs7594727_0w_f	rs7594727	AIMS	97489870	0.908	0.978	0.07063	1	G2
2	snp_rs13012765_0w_r	rs13012765	AncAm	101498174	0.750	0.848	0.09823	1	G2
2	snp_rs1041973_1wa1_r	rs1041973	AIMS	102955468	0.840	0.929	0.08942	1	G2
2	snp_rs1516228_0w_f	rs1516228	AIMS	108274372	0.164	0.512	0.34771	4	G2
2	hmrs1107480_r	rs1107480	AIMS	111953254	0.152	0.029	0.12317	2	G1
2	snp_rs4490198_0w_r	rs4490198	AIMS	115164459	0.335	0.400	0.06507	1	G2
2	snp_rs13014858_0w_r	rs13014858	AncAm	115192890	0.392	0.151	0.24154	3	G1
2	snp_rs4849587_0w_35CT_r	rs4849587	AIMS	118356291	0.112	0.260	0.14888	2	G2
2	snp_rs277554_0w_r	rs277554	AIMS	121631384	0.570	0.084	0.48609	5	G1
2	snp_rs11692789_0w_f	rs11692789	AIMS	123388876	0.454	0.271	0.18324	2	G1
2	snp_rs6739399_0w_f	rs6739399	AIMS	125416607	0.264	0.000	0.26417	3	G1
2	snp_rs881944_0w_f	rs881944	AIMS	127465648	0.580	0.777	0.19694	2	G2
2	snp_rs7567687_1wa1_r	rs7567687	AncAm	130034326	0.473	0.000	0.47342	5	G1
2	snp_rs1955394_0w_r	rs1955394	AIMS	130622888	0.787	0.458	0.32874	4	G1
2	snp_rs2117742_0w_r	rs2117742	AIMS	138048813	0.640	0.803	0.16318	2	G2
2	hmrs11678316_r	rs11678316	AIMS	141751461	0.159	0.219	0.06060	1	G2
2	snp_rs1037266_0w_r	rs1037266	AIMS	142784277	0.293	0.650	0.35612	4	G2
2	snp_rs3768844_0w_f	rs3768844	AIMS	143796694	0.193	0.100	0.09254	1	G1
2	snp_rs7580625_0w_r	rs7580625	AncAm	144146850	0.225	0.793	0.56793	6	G2
2	snp_rs13413446_0w_r	rs13413446	AIMS	145328876	0.194	0.000	0.19378	2	G1
2	snp_rs2381683_0w_f	rs2381683	AncAm	145739556	0.389	0.972	0.58347	6	G2
2	snp_rs10490592_0w_r	rs10490592	AIMS	146931550	0.101	0.042	0.05872	1	G1
2	snp_rs6718445_0w_f	rs6718445	AIMS	148368725	0.601	0.818	0.21755	3	G2
2	snp_rs1975545_0w_r	rs1975545	AIMS	149608697	0.113	0.000	0.11278	2	G1
2	snp_rs6713162_0w_f	rs6713162	AIMS	152496526	0.294	0.132	0.16276	2	G1
2	snp_rs1439638_0w_f	rs1439638	AIMS	154084126	0.736	0.910	0.17374	2	G2
2	snp_rs10497135_0w_r	rs10497135	AncAm	154494454	0.354	0.946	0.59229	6	G2
2	snp_rs10189160_0w_r	rs10189160	AncAm	157012321	0.580	0.039	0.54061	6	G1
2	snp_rs10497191_0w_r	rs10497191	AIMS	158667217	0.200	0.005	0.19481	2	G1
2	snp_rs6759948_0w_f	rs6759948	AncAm	158889593	0.473	0.979	0.50608	6	G2
2	snp_rs4664403_0w_r	rs4664403	AIMS	161933715	0.130	0.175	0.04526	1	G2
2	snp_rs1227131_0w_f	rs1227131	AIMS	168412798	0.449	0.435	0.01397	1	G1
2	snp_rs563694_0w_f	rs563694	AIMS	169774071	0.239	0.000	0.23873	3	G1
2	snp_rs830964_0w_r	rs830964	AncAm	170156880	0.622	0.221	0.40123	5	G1
2	snp_rs7574074_0w_r	rs7574074	AIMS	171548493	0.632	0.192	0.44004	5	G1
2	snp_rs6736440_0w_f	rs6736440	AIMS	174389644	0.317	0.768	0.45063	5	G2
2	snp_rs1005932_0w_r	rs1005932	AIMS	177331710	0.403	0.952	0.54931	6	G2
2	snp_rs2695735_0w_r	rs2695735	Both	178788488	0.327	0.946	0.61984	7	G2
2	snp_rs2627037_0w_f	rs2627037	AIMS	179606538	0.806	0.311	0.49495	5	G1
2	snp_rs4894058_0w_r	rs4894058	AIMS	179738505	0.595	0.387	0.20785	3	G1
2	snp_rs6433782_0w_r	rs6433782	AncAm	180224834	0.591	0.807	0.21586	3	G2
2	snp_rs4894157_0w_f	rs4894157	AIMS	180981724	0.878	0.775	0.10351	2	G1
2	snp_rs7558428_0w_r	rs7558428	AIMS	182018554	0.376	0.043	0.33237	4	G1
2	snp_rs1201151_0w_r	rs1201151	AIMS	189723976	0.066	0.020	0.04527	1	G1
2	snp_rs7568054_0w_r	rs7568054	AncAm	190497896	0.632	0.092	0.53939	6	G1
2	snp_rs9288172_0w_f	rs9288172	AIMS	191250278	0.104	0.023	0.08073	1	G1
2	snp_rs3821236_0w_f	rs3821236	AncAm	191902758	0.741	0.138	0.60274	7	G1
2	snp_rs6710083_0w_r	rs6710083	AIMS	192772592	0.119	0.004	0.11481	2	G1
2	snp_rs10497741_0w_f	rs10497741	AIMS	193920436	0.042	0.000	0.04213	1	G1
2	snp_rs2060798_0w_f	rs2060798	AIMS	195300730	0.182	0.169	0.01298	1	G1
2	snp_rs6434788_0w_r	rs6434788	AIMS	196452537	0.183	0.124	0.05902	1	G1
2	snp_rs6730027_0w_f	rs6730027	AIMS	197876929	0.164	0.056	0.10733	2	G1
2	snp_rs12473502_0w_r	rs12473502	AIMS	199812152	0.613	0.863	0.25026	3	G2
2	snp_rs1569175_0w_r	rs1569175	AIMS	201021954	0.162	0.554	0.39216	4	G2
2	snp_rs3769823_0w_f	rs3769823	AIMS	202122995	0.696	0.238	0.45732	5	G1
2	snp_rs10189499_0w_r	rs10189499	AIMS	203750049	0.560	0.415	0.14524	2	G1
2	snp_rs4675374_0w_f	rs4675374	AIMS	204802578	0.232	0.164	0.06857	1	G1
2	snp_rs1606237_0w_r	rs1606237	AIMS	206247450	0.360	0.000	0.35985	4	G1
2	snp_rs288057_0w_f	rs288057	AIMS	210392711	0.228	0.113	0.11462	2	G1
2	snp_rs1044710_0w_f	rs1044710	AncAm	211297355	0.753	0.185	0.56836	6	G1
2	snp_rs7425448_0w_r	rs7425448	AIMS	212558540	0.056	0.000	0.05581	1	G1
2	snp_rs1317763_0w_r	rs1317763	AIMS	216450655	0.329	0.086	0.24239	3	G1
2	snp_rs1978346_0w_f	rs1978346	AIMS	217561467	0.533	0.816	0.28278	3	G2
2	snp_rs4583451_0w_f	rs4583451	AIMS	221053277	0.172	0.522	0.34986	4	G2
2	snp_rs1955117_0w_r	rs1955117	AIMS	222071265	0.000	0.027	0.02668	1	G2
2	snp_rs825287_0w_f	rs825287	AncAm	222524920	0.478	0.887	0.40872	5	G2
2	snp_rs1250914_0w_r	rs1250914	AncAm	224228654	0.594	0.768	0.17406	2	G2
2	snp_rs2396492_0w_f	rs2396492	AIMS	228586517	0.260	0.023	0.23717	3	G1
2	snp_rs6436883_0w_r	rs6436883	AIMS	230505033	0.451	0.748	0.29706	3	G2
2	snp_rs1667305_0w_f	rs1667305	AIMS	232403829	0.365	0.248	0.11674	2	G1
2	snp_rs11693862_0w_f	rs11693862	AIMS	233999081	0.426	0.296	0.12989	2	G1
2	snp_rs4852054_0w_r	rs4852054	AncAm	240279945	0.494	0.876	0.38179	4	G2
3	snp_rs17047538_0w_f	rs17047538	AIMS	800606	0.178	0.192	0.01401	1	G2
3	snp_rs2727943_0w_f	rs2727943	AncAm	1897973	0.258	0.602	0.34378	4	G2
3	snp_rs10510268_0w_r	rs10510268	AIMS	3564506	0.162	0.000	0.16237	2	G1
3	snp_rs3804989_0w_r	rs3804989	AIMS	4728008	0.449	0.274	0.17419	2	G1
3	snp_rs6802222_0w_r	rs6802222	AIMS	7952801	0.289	0.332	0.04263	1	G2
3	hmrs2581204_f	rs2581204	AIMS	10815485	0.598	0.908	0.31001	4	G2
3	snp_rs7648958_0w_f	rs7648958	AncAm	15621143	0.602	0.295	0.30789	4	G1
3	snp_rs10510446_0w_r	rs10510446	AIMS	16189021	0.368	0.299	0.06937	1	G1
3	snp_rs17043638_0w_f	rs17043638	AncAm	17486643	0.544	0.083	0.46056	5	G1
3	snp_rs12330375_0w_f	rs12330375	AIMS	18391478	0.526	0.941	0.41423	5	G2
3	snp_rs3916092_0w_f	rs3916092	AIMS	19946057	0.919	0.740	0.17925	2	G1
3	snp_rs4858524_0w_f	rs4858524	AIMS	23695943	0.307	0.769	0.46166	5	G2
3	snp_rs1700936_0w_r	rs1700936	AncAm	24327558	0.434	0.135	0.29830	3	G1
3	snp_rs2618111_0w_f	rs2618111	AIMS	27911911	0.070	0.000	0.07017	1	G1
3	snp_rs1517150_0w_r	rs1517150	AIMS	29248536	0.508	0.607	0.09838	1	G2
3	snp_rs744751_1wa0_r	rs744751	AIMS	30735937	0.844	1.000	0.15647	2	G2
3	snp_rs2372321_0w_r	rs2372321	AncAm	31446887	0.524	0.968	0.44416	5	G2
3	snp_rs285327_0w_f	rs285327	AIMS	36314020	0.246	0.120	0.12604	2	G1
3	snp_rs11716902_0w_f	rs11716902	AIMS	38958010	0.355	0.406	0.05063	1	G2
3	snp_rs408499_0w_f	rs408499	AIMS	41093575	0.257	0.086	0.17175	2	G1
3	snp_rs4682869_0w_r	rs4682869	AncAm	42923964	0.503	0.906	0.40310	5	G2
3	snp_rs1013758_0w_f	rs1013758	AIMS	43626375	0.337	0.426	0.08826	1	G2
3	snp_rs2118749_0w_r	rs2118749	AIMS	45572257	0.322	0.000	0.32181	4	G1
3	snp_rs4416372_0w_r	rs4416372	AIMS	51941785	0.943	1.000	0.05692	1	G2
3	snp_rs7427716_0w_r	rs7427716	AIMS	54339576	0.000	0.009	0.00887	1	G2
3	snp_rs9866252_0w_r	rs9866252	AncAm	54897483	0.474	0.017	0.45683	5	G1
3	snp_rs7641315_0w_r	rs7641315	AIMS	55987823	0.000	0.445	0.44547	5	G2
3	snpind_rs9858223_0w_f	rs9858223	AncAm	61238218	0.310	0.919	0.60906	7	G2
3	snp_rs11130841_0w_r	rs11130841	AIMS	61402988	0.508	0.672	0.16435	2	G2
3	snp_rs6795735_0w_f	rs6795735	AIMS	64705365	0.568	1.000	0.43225	5	G2
3	snp_rs1482611_0w_f	rs1482611	AncAm	65121760	0.426	1.000	0.57447	6	G2
3	snp_rs182041_0w_f	rs182041	AIMS	65910218	0.701	0.816	0.11526	2	G2
3	snp_rs805486_0w_f	rs805486	AIMS	70430309	0.376	0.784	0.40808	5	G2
3	snp_rs7617596_0w_f	rs7617596	AncAm	71521494	0.502	0.889	0.38736	4	G2
3	hmrs3749430_f	rs3749430	AIMS	72149119	0.303	0.429	0.12594	2	G2
3	snp_rs6795429_1wa0_f	rs6795429	AncAm	73967610	0.468	0.502	0.03411	1	G2
3	snp_rs6766522_0w_f	rs6766522	AIMS	79077887	0.264	0.179	0.08497	1	G1
3	snp_rs6548616_0w_r	rs6548616	AIMS	79399575	0.329	0.102	0.22722	3	G1
3	snp_rs11127853_0w_f	rs11127853	AIMS	84628861	0.180	0.403	0.22275	3	G2
3	snp_rs9284813_0w_f	rs9284813	AIMS	87152169	0.136	0.127	0.00850	1	G1
3	snp_rs1495824_0w_r	rs1495824	AIMS	88733658	0.402	0.027	0.37504	4	G1
3	snp_rs11713687_0w_r	rs11713687	AIMS	95745701	0.346	0.116	0.22973	3	G1
3	snp_rs2317212_0w_r	rs2317212	Both	97328284	0.460	0.895	0.43487	5	G2
3	snp_rs9289584_0w_f	rs9289584	AIMS	98101101	0.574	0.506	0.06735	1	G1
3	snp_rs6792014_0w_f	rs6792014	AncAm	100244825	0.615	0.250	0.36458	4	G1
3	snp_rs1972991_0w_r	rs1972991	AIMS	100704546	0.495	0.477	0.01834	1	G1
3	hmrs952365_r	rs952365	AncAm	104981251	0.493	0.568	0.07453	1	G2
3	snp_rs7633625_0w_f	rs7633625	AIMS	105069856	0.035	0.013	0.02133	1	G1
3	snp_rs2553955_0w_r	rs2553955	AncAm	107050997	0.483	0.843	0.36016	4	G2
3	snp_rs6808795_0w_f	rs6808795	AIMS	107540714	0.752	0.758	0.00539	1	G2
3	snp_rs746546_0w_r	rs746546	AncAm	108458283	0.430	0.745	0.31514	4	G2
3	snp_rs4535234_0w_f	rs4535234	AIMS	108614492	0.809	0.784	0.02564	1	G1
3	snp_rs10934040_0w_f	rs10934040	AncAm	110153012	0.805	0.873	0.06771	1	G2
3	snp_rs2613964_0w_f	rs2613964	AIMS	112860780	0.561	0.329	0.23204	3	G1
3	snp_rs2062834_0w_f	rs2062834	AIMS	113950127	0.405	0.516	0.11079	2	G2
3	snp_rs7634702_0w_f	rs7634702	AIMS	115329174	0.070	0.000	0.07029	1	G1
3	snp_rs1462843_0w_f	rs1462843	AIMS	116946253	0.225	0.257	0.03178	1	G2
3	snp_rs2869782_0w_r	rs2869782	AIMS	117084425	0.071	0.035	0.03619	1	G1
3	snp_rs1676232_0w_r	rs1676232	AncAm	117234839	0.628	0.891	0.26305	3	G2
3	snp_rs1733302_0w_r	rs1733302	AIMS	120154181	0.269	0.604	0.33489	4	G2
3	snp_rs12629908_0w_35CT_r	rs12629908	AIMS	120522716	0.254	0.549	0.29443	3	G2
3	snp_rs7624614_1wa0_r	rs7624614	AIMS	122303929	0.473	0.203	0.26956	3	G1
3	snp_rs10433406_0w_r	rs10433406	AncAm	124192674	0.224	0.585	0.36061	4	G2
3	snp_rs6800965_0w_r	rs6800965	AIMS	124263881	0.673	0.834	0.16126	2	G2
3	snp_rs4679115_0w_f	rs4679115	AIMS	125997198	0.452	0.299	0.15372	2	G1
3	snp_rs12185961_0w_r	rs12185961	AncAm	127080468	0.618	0.879	0.26118	3	G2
3	snp_rs16827446_0w_f	rs16827446	AIMS	130132202	0.143	0.069	0.07305	1	G1
3	hmrs6808429_f	rs6808429	AIMS	131290291	0.210	0.267	0.05680	1	G2
3	snp_rs4678297_0w_f	rs4678297	AIMS	136907111	0.619	0.789	0.17005	2	G2
3	snp_rs1586861_0w_f	rs1586861	Both	139058496	0.262	1.000	0.73824	8	G2
3	snp_rs13073891_0w_f	rs13073891	AIMS	140140442	0.275	0.784	0.50880	6	G2
3	snp_rs868767_0w_f	rs868767	AIMS	141377330	0.292	0.787	0.49547	5	G2
3	snp_rs9821235_1wa0_f	rs9821235	AIMS	141428703	0.624	0.288	0.33574	4	G1
3	snp_rs9839394_0w_r	rs9839394	AIMS	142951838	0.626	0.427	0.19886	2	G1
3	snp_rs9832471_0w_f	rs9832471	AIMS	143570871	0.058	0.043	0.01447	1	G1
3	snp_rs197858_0w_f	rs197858	AIMS	144072672	0.878	0.953	0.07518	1	G2
3	snp_rs9834043_0w_r	rs9834043	AIMS	145370402	0.439	0.067	0.37199	4	G1
3	snp_rs6788109_0w_r	rs6788109	AIMS	147388753	0.164	0.260	0.09560	1	G2
3	snp_rs7627651_0w_r	rs7627651	AIMS	149922085	0.056	0.000	0.05615	1	G1
3	snp_rs1491975_0w_f	rs1491975	AIMS	151183197	0.278	0.214	0.06405	1	G1
3	snp_rs7639767_0w_r	rs7639767	AIMS	157135595	0.000	0.090	0.08985	1	G2
3	hmrs1714521_f	rs1714521	AIMS	158284861	0.381	0.362	0.01853	1	G1
3	snp_rs6762377_0w_35CT_r	rs6762377	AIMS	159840628	0.860	0.903	0.04318	1	G2
3	snp_rs9865618_0w_r	rs9865618	AIMS	161682714	0.000	0.009	0.00889	1	G2
3	snp_rs1968824_0w_f	rs1968824	AncAm	162261021	0.494	1.000	0.50566	6	G2
3	snp_rs10936383_0w_r	rs10936383	AIMS	163704689	0.211	0.239	0.02790	1	G2
3	snp_rs9851453_0w_r	rs9851453	AIMS	165921816	0.424	0.706	0.28277	3	G2
3	snp_rs9815034_0w_r	rs9815034	AIMS	167497444	0.463	0.379	0.08382	1	G1
3	snp_rs2106124_0w_r	rs2106124	AncAm	169058009	0.698	0.975	0.27642	3	G2
3	snp_rs7613341_0w_f	rs7613341	AIMS	169190907	0.175	0.200	0.02424	1	G2
3	snp_rs6787400_0w_f	rs6787400	AIMS	170357388	0.130	0.358	0.22715	3	G2
3	snp_rs7620616_0w_f	rs7620616	AIMS	171553796	0.172	0.158	0.01397	1	G1
3	snp_rs635255_0w_r	rs635255	AIMS	173198359	0.391	0.144	0.24726	3	G1
3	snp_rs1515596_0w_f	rs1515596	AncAm	174298999	0.363	0.874	0.51195	6	G2
3	snp_rs7636818_0w_f	rs7636818	AIMS	179022115	0.129	0.105	0.02386	1	G1
3	snp_rs12635682_0w_f	rs12635682	AIMS	180524108	0.167	0.513	0.34673	4	G2
3	snp_rs10937074_0w_r	rs10937074	AIMS	181637333	0.629	0.241	0.38788	4	G1
3	snp_rs4859259_0w_f	rs4859259	AIMS	182664547	0.116	0.676	0.56067	6	G2
3	snp_rs6443987_0w_r	rs6443987	AncAm	184406997	0.431	0.821	0.39041	4	G2
3	snp_rs9872799_0w_r	rs9872799	AIMS	184428903	0.732	0.091	0.64121	7	G1
3	snp_rs9867846_0w_f	rs9867846	AIMS	185746890	0.247	0.663	0.41602	5	G2
3	snp_rs6444127_0w_f	rs6444127	AncAm	186143744	0.556	0.033	0.52321	6	G1
3	snp_rs6775595_0w_f	rs6775595	AIMS	189133577	0.801	0.592	0.20869	3	G1
3	snp_rs10513852_0w_f	rs10513852	AIMS	190167533	0.956	0.948	0.00873	1	G1
3	snp_rs7642497_0w_f	rs7642497	AncAm	190207425	0.590	0.931	0.34036	4	G2
3	snp_rs4686599_0w_f	rs4686599	AIMS	191845112	0.132	0.467	0.33491	4	G2
3	snp_rs7642439_0w_r	rs7642439	AIMS	192949982	0.086	0.016	0.06958	1	G1
4	snp_rs7437940_0w_r	rs7437940	AncAm	7887500	0.339	0.020	0.31866	4	G1
4	snp_rs959190_0w_f	rs959190	AIMS	10872741	0.078	0.110	0.03254	1	G2
4	snp_rs1019661_0w_r	rs1019661	AIMS	14188591	0.151	0.091	0.06019	1	G1
4	snp_rs6449134_0w_f	rs6449134	AncAm	15380517	0.689	0.974	0.28500	3	G2
4	snp_rs10001565_0w_f	rs10001565	AIMS	15722573	0.061	0.059	0.00269	1	G1
4	snp_rs3846382_0w_f	rs3846382	AIMS	19609939	0.175	0.485	0.31000	4	G2
4	snp_rs12505641_0w_f	rs12505641	AncAm	23488892	0.402	0.764	0.36135	4	G2
4	snp_rs1395221_0w_r	rs1395221	AIMS	24626903	0.827	0.796	0.03093	1	G1
4	snp_rs6831024_0w_f	rs6831024	AIMS	25863375	0.521	0.291	0.22943	3	G1
4	snp_rs9884706_0w_f	rs9884706	AIMS	32632087	0.133	0.102	0.03085	1	G1
4	snp_rs7664105_0w_f	rs7664105	AIMS	33935750	0.081	0.188	0.10662	2	G2
4	snp_rs6817183_0w_f	rs6817183	AIMS	34498265	0.892	0.766	0.12513	2	G1
4	snp_rs1655069_1wa0_f	rs1655069	AIMS	35923989	0.625	0.828	0.20263	3	G2
4	snp_rs4832844_0w_r	rs4832844	AIMS	36948351	0.605	0.522	0.08262	1	G1
4	snp_rs11725412_0w_f	rs11725412	Both	38277754	0.563	0.178	0.38483	4	G1
4	snp_rs12186184_0w_r	rs12186184	AIMS	41297430	0.353	0.405	0.05289	1	G2
4	snp_rs10026397_0w_r	rs10026397	AIMS	41565887	0.806	0.848	0.04252	1	G2
4	snp_rs2120041_0w_f	rs2120041	AIMS	43267971	0.014	0.000	0.01394	1	G1
4	snp_rs1389037_0w_f	rs1389037	AIMS	45794893	0.598	0.491	0.10671	2	G1
4	snp_rs10938462_0w_f	rs10938462	AncAm	46890143	0.803	0.293	0.51041	6	G1
4	snp_rs6832891_0w_r	rs6832891	AIMS	55056644	0.531	0.266	0.26466	3	G1
4	snp_rs10031105_0w_f	rs10031105	AIMS	56609872	0.111	0.063	0.04749	1	G1
4	snp_rs7679852_0w_f	rs7679852	AncAm	56683846	0.553	0.000	0.55318	6	G1
4	hmrs10222715_f	rs10222715	AIMS	57614160	0.472	0.553	0.08141	1	G2
4	snp_rs2124236_0w_f	rs2124236	AIMS	61706356	0.530	0.048	0.48203	5	G1
4	snp_rs10866111_0w_r	rs10866111	AncAm	61900046	0.593	0.000	0.59279	6	G1
4	snp_rs6843214_0w_r	rs6843214	AIMS	63354303	0.390	0.072	0.31857	4	G1
4	snp_rs6855459_0w_r	rs6855459	AIMS	64404377	0.530	0.364	0.16566	2	G1
4	snp_rs1440949_0w_r	rs1440949	AIMS	65883560	0.458	0.545	0.08675	1	G2
4	snp_rs11724259_0w_r	rs11724259	AIMS	68159587	0.221	0.171	0.05070	1	G1
4	snp_rs4261956_0w_f	rs4261956	AIMS	72341864	0.367	0.175	0.19164	2	G1
4	snp_rs1369093_0w_35AG_r	rs1369093	AIMS	73245191	0.826	0.193	0.63255	7	G1
4	snp_rs11722134_0w_r	rs11722134	AIMS	73556348	0.232	0.039	0.19308	2	G1
4	snp_rs3923243_0w_r	rs3923243	AIMS	75579453	0.275	0.000	0.27456	3	G1
4	snp_rs10032423_0w_f	rs10032423	AncAm	77140733	0.471	0.018	0.45343	5	G1
4	snp_rs3733242_0w_f	rs3733242	AIMS	77675505	0.569	0.474	0.09475	1	G1
4	snp_rs2055493_0w_f	rs2055493	AIMS	78981919	0.244	0.209	0.03515	1	G1
4	snp_rs1455313_0w_r	rs1455313	AIMS	80121894	0.249	0.277	0.02764	1	G2
4	snp_rs10516652_0w_f	rs10516652	AIMS	82166904	0.153	0.453	0.30011	4	G2
4	snp_rs6820697_0w_f	rs6820697	AIMS	84800475	0.431	0.426	0.00583	1	G1
4	snp_rs385194_0w_r	rs385194	AIMS	85309078	0.636	0.782	0.14607	2	G2
4	snp_rs2869703_0w_f	rs2869703	AIMS	88626278	0.409	0.451	0.04181	1	G2
4	snp_rs1343921_0w_r	rs1343921	AIMS	89947462	0.353	0.171	0.18207	2	G1
4	snp_rs1355993_0w_r	rs1355993	AIMS	91661745	0.184	0.062	0.12194	2	G1
4	snp_rs1485989_0w_f	rs1485989	AIMS	92758836	0.188	0.673	0.48507	5	G2
4	snp_rs7669241_0w_f	rs7669241	AIMS	100430924	0.094	0.109	0.01535	1	G2
4	snp_rs2850971_0w_r	rs2850971	AIMS	102184626	0.463	0.709	0.24634	3	G2
4	hmrs13152048_f	rs13152048	AIMS	103275840	0.229	0.015	0.21373	3	G1
4	snp_rs7657799_0w_f	rs7657799	AIMS	105375423	0.070	0.000	0.06950	1	G1
4	snp_rs7682418_0w_r	rs7682418	AIMS	105428417	0.749	0.983	0.23352	3	G2
4	snp_rs4235415_0w_f	rs4235415	AIMS	106593850	0.193	0.162	0.03140	1	G1
4	snp_rs1056079_1wa0_f	rs1056079	AIMS	108222771	0.272	0.932	0.66060	7	G2
4	hmrs2194860_f	rs2194860	AIMS	109948511	0.190	0.954	0.76329	8	G2
4	snp_rs1448817_0w_r	rs1448817	AIMS	111641053	0.537	0.333	0.20389	3	G1
4	snp_rs4269197_0w_f	rs4269197	AncAm	115638209	0.342	0.757	0.41504	5	G2
4	snp_rs4128688_0w_r	rs4128688	AIMS	118788592	0.793	0.607	0.18576	2	G1
4	snp_rs10518336_0w_f	rs10518336	AIMS	120522934	0.721	0.635	0.08604	1	G1
4	snp_rs692157_0w_r	rs692157	AIMS	122382834	0.373	0.474	0.10066	2	G2
4	snp_rs13115814_0w_f	rs13115814	AIMS	124545508	0.170	0.123	0.04632	1	G1
4	snp_rs3956574_0w_f	rs3956574	AIMS	126039335	0.321	0.605	0.28365	3	G2
4	snp_rs2391576_0w_f	rs2391576	AIMS	130927461	0.227	0.000	0.22700	3	G1
4	snp_rs2419533_0w_f	rs2419533	AIMS	133589514	0.609	0.243	0.36560	4	G1
4	snp_rs1451118_0w_r	rs1451118	AIMS	137106966	0.443	0.545	0.10135	2	G2
4	snp_rs10519410_0w_f	rs10519410	AIMS	138134581	0.185	0.000	0.18498	2	G1
4	snp_rs1112828_0w_f	rs1112828	AIMS	139956970	0.643	0.111	0.53164	6	G1
4	snp_rs706343_0w_r	rs706343	AIMS	140548713	0.433	0.811	0.37831	4	G2
4	snp_rs3775605_0w_r	rs3775605	AIMS	142968081	0.387	0.111	0.27632	3	G1
4	snp_rs1507500_1wa0_f	rs1507500	AIMS	147993702	0.167	0.303	0.13617	2	G2
4	snp_rs6826460_0w_r	rs6826460	AncAm	149605653	0.679	0.173	0.50585	6	G1
4	snp_rs6852150_0w_f	rs6852150	AIMS	150818662	0.070	0.000	0.07019	1	G1
4	snp_rs7663659_0w_f	rs7663659	AIMS	152175168	0.501	0.000	0.50058	6	G1
4	snp_rs11935573_0w_f	rs11935573	AIMS	155241572	0.642	0.784	0.14245	2	G2
4	snp_rs7677803_0w_f	rs7677803	AncAm	156409087	0.437	0.878	0.44190	5	G2
4	snp_rs10517660_0w_f	rs10517660	AIMS	157842396	0.547	0.612	0.06467	1	G2
4	snp_rs9762089_0w_f	rs9762089	AncAm	160630642	0.360	0.759	0.39861	4	G2
4	snp_rs6827968_0w_r	rs6827968	AncAm	161399652	0.587	0.176	0.41077	5	G1
4	snp_rs1492468_0w_r	rs1492468	AIMS	163030012	0.180	0.073	0.10714	2	G1
4	snp_rs1870477_0w_f	rs1870477	AncAm	166605228	0.313	0.147	0.16552	2	G1
4	snp_rs703887_0w_r	rs703887	AIMS	167136508	0.145	0.316	0.17081	2	G2
4	snp_rs6552223_0w_f	rs6552223	AncAm	167694282	0.291	0.111	0.17945	2	G1
4	snp_rs3811813_0w_f	rs3811813	AIMS	170037572	0.074	0.015	0.05941	1	G1
4	snp_rs744724_0w_r	rs744724	AIMS	174459823	0.169	0.115	0.05493	1	G1
4	snp_rs11730565_0w_f	rs11730565	AIMS	176326439	0.014	0.000	0.01416	1	G1
4	snp_rs12715977_0w_f	rs12715977	AncAm	177495414	0.518	0.097	0.42153	5	G1
4	snp_rs6552216_0w_f	rs6552216	AncAm	178112004	0.663	0.268	0.39582	4	G1
4	snp_rs3911610_0w_r	rs3911610	AncAm	180472174	0.792	0.450	0.34212	4	G1
4	snp_rs7673020_unw_f	rs7673020	AIMS	180962285	0.153	0.063	0.09074	1	G1
4	snp_rs10012103_0w_f	rs10012103	AncAm	184531409	0.499	0.127	0.37191	4	G1
4	snp_rs1435442_0w_r	rs1435442	AIMS	184817202	0.213	0.000	0.21281	3	G1
4	snp_rs5018568_0w_f	rs5018568	AncAm	186617593	0.677	0.176	0.50106	6	G1
4	snp_rs2165666_0w_f	rs2165666	AIMS	187461706	0.567	0.582	0.01427	1	G2
4	snp_rs11734306_0w_r	rs11734306	AncAm	188973194	0.370	0.130	0.24070	3	G1
5	snp_rs378257_0w_r	rs378257	AIMS	2518738	0.675	0.859	0.18368	2	G2
5	snp_rs698147_0w_r	rs698147	AIMS	3513485	0.582	0.385	0.19758	2	G1
5	hmrs26880_f	rs26880	AIMS	4905892	0.546	0.488	0.05802	1	G1
5	snp_rs10037656_0w_f	rs10037656	AncAm	5183783	0.441	0.860	0.41933	5	G2
5	snp_rs7713650_0w_r	rs7713650	AIMS	7169448	0.121	0.031	0.09002	1	G1
5	snp_rs11134354_0w_f	rs11134354	AncAm	9421794	0.618	0.213	0.40498	5	G1
5	hmrs6555619_f	rs6555619	AIMS	9896146	0.202	0.121	0.08107	1	G1
5	snp_rs31887_0w_r	rs31887	AIMS	11423592	0.026	0.064	0.03814	1	G2
5	snp_rs10513144_0w_r	rs10513144	AIMS	13633968	0.351	0.151	0.19972	2	G1
5	snp_rs4235556_0w_f	rs4235556	AIMS	15738017	0.689	0.489	0.20007	3	G1
5	snp_rs1872004_0w_f	rs1872004	AIMS	16807786	0.158	0.326	0.16821	2	G2
5	snp_rs1155040_0w_f	rs1155040	AIMS	19814107	0.439	0.474	0.03546	1	G2
5	snp_rs7714385_1wa1_f	rs7714385	AIMS	24500645	0.338	0.450	0.11191	2	G2
5	snp_rs1565873_0w_f	rs1565873	AIMS	26374931	0.313	0.308	0.00583	1	G1
5	snp_rs4382127_0w_f	rs4382127	AncAm	27252466	0.506	0.169	0.33693	4	G1
5	snp_rs16896876_0w_r	rs16896876	AIMS	27523340	0.793	0.874	0.08138	1	G2
5	snp_rs1366363_0w_f	rs1366363	AncAm	29949620	0.667	0.242	0.42540	5	G1
5	snp_rs1051489_0w_f	rs1051489	AIMS	32400266	0.307	0.312	0.00476	1	G2
5	hmrs35391_f	rs35391	AncAm	33955673	0.341	0.831	0.49022	5	G2
5	snp_rs9292587_0w_f	rs9292587	AIMS	35364788	0.731	0.983	0.25237	3	G2
5	snp_rs4418129_0w_r	rs4418129	AIMS	36398024	0.303	0.045	0.25809	3	G1
5	snp_rs2886725_0w_r	rs2886725	AncAm	37770836	0.628	0.328	0.29965	3	G1
5	snp_rs696737_0w_f	rs696737	AIMS	39195423	0.000	0.036	0.03565	1	G2
5	snp_rs353372_0w_f	rs353372	AncAm	40226919	0.489	0.560	0.07156	1	G2
5	snp_rs1553578_0w_f	rs1553578	AIMS	40669184	0.424	0.762	0.33752	4	G2
5	snp_rs10512806_0w_f	rs10512806	AIMS	42838556	0.000	0.062	0.06224	1	G2
5	hmrs6451722_r	rs6451722	AIMS	43711378	0.928	0.920	0.00858	1	G1
5	snp_rs6875400_0w_f	rs6875400	AIMS	43862694	0.000	0.000	0.00000	1	0.000
5	snp_rs13340341_0w_f	rs13340341	AIMS	45860936	0.365	0.434	0.06844	1	G2
5	snp_rs1039797_0w_f	rs1039797	AIMS	49651872	0.412	0.441	0.02895	1	G2
5	snp_rs4865695_0w_f	rs4865695	AIMS	51199109	0.541	0.527	0.01395	1	G1
5	snp_rs2269225_0w_f	rs2269225	AIMS	54276104	0.085	0.354	0.26939	3	G2
5	snp_rs7718757_0w_r	rs7718757	AIMS	56930116	0.930	1.000	0.07003	1	G2
5	snp_rs1816392_0w_f	rs1816392	AncAm	57147676	0.162	0.714	0.55223	6	G2
5	snp_rs1533019_0w_r	rs1533019	AIMS	59307813	0.182	0.007	0.17528	2	G1
5	snp_rs6875401_0w_r	rs6875401	AncAm	62456005	0.285	0.087	0.19775	2	G1
5	snp_rs10054295_0w_f	rs10054295	AIMS	63035923	0.131	0.068	0.06378	1	G1
5	snp_rs29460_0w_r	rs29460	AIMS	65534403	0.309	0.239	0.06927	1	G1
5	snp_rs2888306_0w_r	rs2888306	AIMS	67412484	0.340	0.611	0.27090	3	G2
5	snp_rs464923_0w_f	rs464923	AIMS	72177890	0.149	0.041	0.10719	2	G1
5	snp_rs4479825_0w_f	rs4479825	AncAm	73527328	0.277	0.664	0.38676	4	G2
5	snp_rs1001935_0w_r	rs1001935	AIMS	75040180	0.099	0.150	0.05097	1	G2
5	snp_rs2279095_0w_f	rs2279095	AIMS	76709426	0.282	0.168	0.11392	2	G1
5	snp_rs234688_0w_f	rs234688	AIMS	78199454	0.423	0.282	0.14076	2	G1
5	snp_rs1373967_0w_f	rs1373967	AIMS	80216556	0.212	0.731	0.51873	6	G2
5	snp_rs173686_0w_f	rs173686	AIMS	82811500	0.578	0.512	0.06609	1	G1
5	snp_rs6452576_0w_r	rs6452576	AIMS	83981802	0.144	0.353	0.20865	3	G2
5	snp_rs2974061_0w_f	rs2974061	AIMS	85227164	0.498	0.493	0.00512	1	G1
5	snp_rs9293471_0w_f	rs9293471	AncAm	85960197	0.528	0.800	0.27253	3	G2
5	snp_rs6452788_0w_f	rs6452788	AIMS	87712913	0.707	0.492	0.21477	3	G1
5	snp_rs1852723_0w_r	rs1852723	AIMS	88760511	0.388	0.791	0.40324	5	G2
5	snp_rs16869392_0w_r	rs16869392	AncAm	90350998	0.197	0.692	0.49432	5	G2
5	snp_rs7725232_0w_f	rs7725232	AIMS	92574893	0.099	0.062	0.03685	1	G1
5	snp_rs9127_0w_f	rs9127	AIMS	96372165	0.372	0.138	0.23378	3	G1
5	snp_rs10515276_0w_f	rs10515276	AIMS	97836794	0.040	0.020	0.02021	1	G1
5	snp_rs174015_1wa0_f	rs174015	AIMS	102131967	0.635	0.830	0.19524	2	G2
5	snp_rs17157450_0w_r	rs17157450	Both	104356919	0.165	0.878	0.71281	8	G2
5	hmrs12514674_r	rs12514674	AIMS	107159087	0.267	0.035	0.23192	3	G1
5	snp_rs13354224_0w_f	rs13354224	AIMS	108820735	0.086	0.025	0.06114	1	G1
5	snp_rs4339398_0w_f	rs4339398	AIMS	109353981	0.887	0.983	0.09523	1	G2
5	snp_rs13358140_0w_f	rs13358140	AIMS	112016578	0.051	0.056	0.00550	1	G2
5	snp_rs13186060_0w_r	rs13186060	AIMS	113207613	0.296	0.005	0.29140	3	G1
5	snp_rs10071490_0w_r	rs10071490	AncAm	113940187	0.821	0.270	0.55016	6	G1
5	snp_rs10519406_0w_r	rs10519406	AIMS	114691287	0.284	0.459	0.17492	2	G2
5	snp_rs3797569_0w_f	rs3797569	AncAm	115208843	0.153	0.756	0.60309	7	G2
5	snp_rs13356416_0w_f	rs13356416	AIMS	116439083	0.072	0.000	0.07162	1	G1
5	snp_rs10079352_0w_f	rs10079352	Both	117494640	0.536	0.934	0.39851	4	G2
5	snp_rs10056223_0w_f	rs10056223	AIMS	117770863	0.120	0.075	0.04434	1	G1
5	snp_rs3909319_0w_f	rs3909319	AIMS	120703917	0.151	0.162	0.01140	1	G2
5	snp_rs6863510_0w_f	rs6863510	AIMS	124184547	0.873	0.930	0.05735	1	G2
5	snp_rs3909885_0w_r	rs3909885	AIMS	126918504	0.203	0.646	0.44316	5	G2
5	snp_rs6866231_0w_f	rs6866231	AIMS	128803345	0.000	0.223	0.22258	3	G2
5	snp_rs803137_0w_f	rs803137	AncAm	132132647	0.353	0.930	0.57712	6	G2
5	hmrs4958148_r	rs4958148	AIMS	133072525	0.388	0.305	0.08360	1	G1
5	snp_rs346650_0w_r	rs346650	AIMS	135724921	0.209	0.262	0.05278	1	G2
5	snp_rs1528961_0w_f	rs1528961	AIMS	136793148	0.143	0.631	0.48820	5	G2
5	snp_rs7732591_0w_r	rs7732591	AIMS	142125177	0.147	0.429	0.28188	3	G2
5	snp_rs7721458_0w_f	rs7721458	AIMS	142863240	0.588	0.622	0.03459	1	G2
5	snp_rs10515553_0w_r	rs10515553	AIMS	145038942	0.312	0.000	0.31235	4	G1
5	snp_rs4913050_0w_r	rs4913050	AncAm	145282109	0.350	0.461	0.11116	2	G2
5	snp_rs13180336_0w_r	rs13180336	AncAm	148868169	0.519	0.032	0.48688	5	G1
5	snp_rs2615152_0w_r	rs2615152	AIMS	152696107	0.057	0.124	0.06642	1	G2
5	snp_rs1461237_0w_f	rs1461237	AncAm	153238323	0.701	0.146	0.55549	6	G1
5	snp_rs1864956_0w_f	rs1864956	AncAm	155326812	0.500	0.079	0.42127	5	G1
5	hmrs1650576_r	rs1650576	AIMS	157655044	0.693	0.609	0.08463	1	G1
5	snp_rs1038704_0w_f	rs1038704	AIMS	160551087	0.111	0.054	0.05632	1	G1
5	snp_rs2964356_0w_r	rs2964356	AIMS	162626996	0.432	0.336	0.09571	1	G1
5	snp_rs10066478_0w_f	rs10066478	AncAm	164370690	0.518	0.328	0.18996	2	G1
5	snp_rs1422680_0w_r	rs1422680	AIMS	164666384	0.726	0.890	0.16375	2	G2
5	snp_rs1500127_0w_f	rs1500127	AIMS	165739982	0.142	0.571	0.42948	5	G2
5	snp_rs10516076_0w_r	rs10516076	AIMS	169896090	0.000	0.044	0.04434	1	G2
5	snp_rs171718_0w_f	rs171718	AIMS	171143166	0.392	0.577	0.18525	2	G2
5	snp_rs4868204_0w_f	rs4868204	AIMS	172204629	0.221	0.339	0.11802	2	G2
5	snp_rs2135053_0w_f	rs2135053	AIMS	174465510	0.517	0.355	0.16167	2	G1
5	snp_rs7727897_0w_f	rs7727897	AIMS	177047009	0.151	0.206	0.05462	1	G2
5	snp_rs9329079_0w_f	rs9329079	AncAm	178936722	0.508	0.144	0.36343	4	G1
5	snp_rs248327_0w_r	rs248327	AIMS	179377650	0.425	0.503	0.07771	1	G2
6	snp_rs1933652_0w_r	rs1933652	AIMS	741043	0.124	0.108	0.01583	1	G1
6	snp_rs2326106_0w_r	rs2326106	AIMS	2989582	0.822	0.767	0.05500	1	G1
6	snp_rs6939368_0w_f	rs6939368	AIMS	4683536	0.020	0.103	0.08362	1	G2
6	snp_rs3827784_0w_r	rs3827784	AncAm	6642405	0.231	0.451	0.22006	3	G2
6	snp_rs4960257_0w_r	rs4960257	AIMS	6865244	0.437	0.434	0.00323	1	G1
6	snp_rs2876167_0w_r	rs2876167	AIMS	9481372	0.111	0.454	0.34285	4	G2
6	snp_rs6910232_0w_r	rs6910232	AncAm	10902439	0.450	0.319	0.13142	2	G1
6	snp_rs12662518_0w_r	rs12662518	AIMS	12219785	0.182	0.000	0.18170	2	G1
6	snp_rs1992387_1wa1_r	rs1992387	AIMS	13431314	0.092	0.022	0.06975	1	G1
6	snp_rs13199398_0w_r	rs13199398	AncAm	15713820	0.458	0.129	0.32910	4	G1
6	snp_rs7745461_0w_f	rs7745461	AIMS	21911616	0.754	0.343	0.41081	5	G1
6	snp_rs1928168_0w_f	rs1928168	AIMS	22017738	0.360	0.120	0.24003	3	G1
6	snp_rs7753473_0w_f	rs7753473	AncAm	22726200	0.309	0.142	0.16674	2	G1
6	snp_rs9379502_0w_f	rs9379502	AIMS	23358239	0.410	0.299	0.11054	2	G1
6	snp_rs6918101_0w_f	rs6918101	AIMS	25027288	0.358	0.324	0.03398	1	G1
6	snp_rs2747454_0w_f	rs2747454	AIMS	29655113	0.318	0.376	0.05845	1	G2
6	snp_rs12660883_0w_f	rs12660883	AIMS	30764420	0.442	0.537	0.09505	1	G2
6	snp_rs382259_0w_f	rs382259	AIMS	32209027	0.590	0.851	0.26065	3	G2
6	hmrs3097669_r	rs3097669	AncAm	33023792	0.685	0.778	0.09294	1	G2
6	snp_rs804847_0w_f	rs804847	AIMS	37547513	0.428	0.474	0.04584	1	G2
6	snp_rs9357342_0w_f	rs9357342	AIMS	40797236	0.190	0.280	0.08979	1	G2
6	snp_rs7755400_0w_r	rs7755400	AIMS	42197916	0.351	0.280	0.07137	1	G1
6	snp_rs4714598_0w_f	rs4714598	AncAm	42282393	0.496	0.973	0.47701	5	G2
6	snp_rs9357427_0w_f	rs9357427	AIMS	43977535	0.199	0.077	0.12192	2	G1
6	snp_rs12524885_0w_r	rs12524885	AIMS	45836069	0.260	0.403	0.14369	2	G2
6	snp_rs2397060_0w_f	rs2397060	AIMS	51611470	0.117	0.103	0.01362	1	G1
6	snp_rs788510_0w_r	rs788510	AIMS	51705624	0.583	0.803	0.22012	3	G2
6	snp_rs9357726_0w_f	rs9357726	AncAm	52025693	0.430	0.955	0.52423	6	G2
6	snp_rs9367532_0w_f	rs9367532	AncAm	53310839	0.168	0.871	0.70258	8	G2
6	snp_rs1883632_0w_f	rs1883632	AIMS	53780432	0.311	0.460	0.14904	2	G2
6	snp_rs721423_0w_r	rs721423	AIMS	63034767	0.051	0.184	0.13348	2	G2
6	snp_rs2055172_0w_r	rs2055172	AIMS	65484758	0.186	0.140	0.04605	1	G1
6	snp_rs701662_0w_r	rs701662	AIMS	68890028	0.115	0.158	0.04324	1	G2
6	snp_rs3806033_0w_f	rs3806033	AIMS	70828336	0.411	0.778	0.36696	4	G2
6	snp_rs2057155_0w_r	rs2057155	AIMS	80448393	0.343	0.298	0.04551	1	G1
6	snp_rs7761777_0w_r	rs7761777	AncAm	81092467	0.528	0.002	0.52671	6	G1
6	snp_rs1932215_0w_f	rs1932215	AIMS	83559409	0.217	0.063	0.15432	2	G1
6	snp_rs9359682_0w_r	rs9359682	AIMS	86896768	0.314	0.227	0.08717	1	G1
6	snp_rs192655_0w_f	rs192655	AIMS	90518278	0.358	0.500	0.14243	2	G2
6	snp_rs4610536_0w_f	rs4610536	AIMS	91914865	0.726	0.756	0.03007	1	G2
6	snp_rs1391502_0w_f	rs1391502	AncAm	92615457	0.301	0.901	0.60016	7	G2
6	snp_rs794672_0w_f	rs794672	AIMS	95458317	0.750	0.884	0.13396	2	G2
6	snp_rs9404150_0w_f	rs9404150	AncAm	96474372	0.147	0.680	0.53392	6	G2
6	snp_rs746810_0w_f	rs746810	AIMS	96490089	0.251	0.241	0.00976	1	G1
6	snp_rs9320598_0w_f	rs9320598	AncAm	98019080	0.059	0.767	0.70819	8	G2
6	snp_rs551185_0w_f	rs551185	AIMS	100106323	0.249	0.079	0.16991	2	G1
6	snp_rs7752055_0w_f	rs7752055	AIMS	105960712	0.093	0.048	0.04490	1	G1
6	snp_rs2001144_0w_f	rs2001144	AIMS	108407662	0.182	0.487	0.30476	4	G2
6	snp_rs539630_0w_f	rs539630	AIMS	108610641	0.182	0.364	0.18175	2	G2
6	snp_rs9320326_0w_r	rs9320326	AIMS	110657142	0.233	0.305	0.07170	1	G2
6	snp_rs4947199_0w_r	rs4947199	AIMS	113013481	0.863	0.704	0.15886	2	G1
6	snp_rs9384878_0w_f	rs9384878	Both	113975118	0.490	0.124	0.36592	4	G1
6	snp_rs352092_0w_r	rs352092	AIMS	114240685	0.698	0.605	0.09226	1	G1
6	snp_rs7738597_0w_r	rs7738597	AIMS	118263296	0.428	0.002	0.42610	5	G1
6	snp_rs11750983_0w_f	rs11750983	AIMS	121528926	0.061	0.006	0.05530	1	G1
6	snp_rs17083435_0w_f	rs17083435	AncAm	121662909	0.319	0.720	0.40191	5	G2
6	snp_rs13200297_0w_r	rs13200297	AIMS	123251303	0.185	0.092	0.09325	1	G1
6	snp_rs802228_0w_f	rs802228	AIMS	124526649	0.569	0.367	0.20236	3	G1
6	snp_rs6906366_0w_r	rs6906366	AIMS	126284213	0.000	0.000	0.00000	1	0.000
6	snp_rs9491563_0w_f	rs9491563	AncAm	126449345	0.260	0.235	0.02516	1	G1
6	snp_rs802726_0w_f	rs802726	AIMS	128281861	0.764	0.669	0.09472	1	G1
6	snp_rs2451688_0w_f	rs2451688	AIMS	129646565	0.014	0.044	0.02982	1	G2
6	snp_rs6940184_0w_r	rs6940184	AIMS	132257444	0.280	0.142	0.13718	2	G1
6	snp_rs9689446_0w_35CT_f	rs9689446	AIMS	134461443	0.153	0.000	0.15268	2	G1
6	hmrs6570569_f	rs6570569	AncAm	143939639	0.334	0.779	0.44501	5	G2
6	snp_rs4895642_0w_f	rs4895642	AncAm	144758799	0.298	0.967	0.66953	7	G2
6	snp_rs1406045_0w_f	rs1406045	AIMS	145004411	0.456	0.000	0.45589	5	G1
6	snp_rs4243474_0w_r	rs4243474	AncAm	145096340	0.582	0.020	0.56255	6	G1
6	hmrs11753794_f	rs11753794	AIMS	151418320	0.372	0.341	0.03084	1	G1
6	snp_rs7752591_0w_r	rs7752591	AncAm	151947068	0.519	0.002	0.51719	6	G1
6	hmrs2352423_r	rs2352423	AIMS	155540230	0.261	0.083	0.17744	2	G1
6	snp_rs324368_0w_f	rs324368	AncAm	155642564	0.436	0.919	0.48342	5	G2
6	snp_rs438657_0w_f	rs438657	AncAm	159948959	0.566	0.111	0.45493	5	G1
6	snp_rs9457827_0w_r	rs9457827	AIMS	160503656	0.260	0.423	0.16296	2	G2
6	hmrs577876_r	rs577876	AIMS	161923155	0.240	0.132	0.10785	2	G1
6	snp_rs10428822_0w_35AC_r	rs10428822	AIMS	166603765	0.000	0.161	0.16063	2	G2
6	snp_rs9364195_0w_r	rs9364195	AIMS	169410675	0.853	0.969	0.11608	2	G2
6	snp_rs7769343_0w_r	rs7769343	AIMS	170517906	0.142	0.052	0.08993	1	G1
7	hmrs4281072_r	rs4281072	AIMS	54183	0.201	0.514	0.31303	4	G2
7	snp_rs2041362_0w_f	rs2041362	AIMS	8044483	0.835	0.643	0.19205	2	G1
7	snp_rs2189947_0w_r	rs2189947	AIMS	10319014	0.400	0.172	0.22884	3	G1
7	snp_rs739773_0w_r	rs739773	AIMS	11394610	0.126	0.000	0.12565	2	G1
7	snp_rs731257_0w_r	rs731257	Both	12669251	0.627	0.167	0.45982	5	G1
7	snp_rs2080161_0w_r	rs2080161	Both	13331150	0.256	0.916	0.66012	7	G2
7	snp_rs10267453_0w_r	rs10267453	AIMS	14133305	0.618	0.531	0.08706	1	G1
7	snp_rs697518_0w_f	rs697518	AIMS	16759108	0.112	0.000	0.11206	2	G1
7	snp_rs2529015_0w_f	rs2529015	AIMS	20326586	0.112	0.000	0.11222	2	G1
7	snp_rs1320800_0w_f	rs1320800	AncAm	21991532	0.792	0.332	0.46018	5	G1
7	snp_rs2905308_0w_f	rs2905308	AIMS	22648194	0.592	0.788	0.19574	2	G2
7	hmrs10951140_f	rs10951140	AIMS	26531720	0.182	0.846	0.66334	7	G2
7	snp_rs3793306_0w_f	rs3793306	AncAm	29314083	0.260	0.876	0.61554	7	G2
7	snp_rs1362352_0w_f	rs1362352	AIMS	29594774	0.339	0.425	0.08614	1	G2
7	snp_rs2302019_0w_f	rs2302019	AIMS	31003423	0.623	0.680	0.05688	1	G2
7	snp_rs7794503_0w_r	rs7794503	AIMS	31048898	0.216	0.004	0.21201	3	G1
7	snp_rs6947433_0w_r	rs6947433	AIMS	32176431	0.267	0.000	0.26651	3	G1
7	snp_rs17170516_0w_f	rs17170516	Both	32818759	0.210	0.906	0.69665	7	G2
7	snp_rs3801305_0w_r	rs3801305	AIMS	36480876	0.088	0.430	0.34228	4	G2
7	snp_rs2214875_0w_f	rs2214875	AIMS	41793043	0.158	0.193	0.03542	1	G2
7	snp_rs6948541_0w_r	rs6948541	AIMS	48396976	0.056	0.360	0.30403	4	G2
7	snp_rs933360_0w_r	rs933360	AIMS	50758245	0.274	0.368	0.09372	1	G2
7	snp_rs17151293_0w_f	rs17151293	AncAm	51973256	0.450	0.881	0.43161	5	G2
7	snp_rs17135491_0w_f	rs17135491	AIMS	53105600	0.030	0.328	0.29806	3	G2
7	snp_rs17564002_0w_f	rs17564002	AIMS	54795837	0.496	0.892	0.39578	4	G2
7	snp_rs10239854_0w_f	rs10239854	AncAm	55714282	0.456	0.155	0.30122	4	G1
7	snp_rs4718701_0w_r	rs4718701	AIMS	67515801	0.049	0.103	0.05404	1	G2
7	snp_rs7811944_0w_f	rs7811944	AIMS	68724055	0.799	0.501	0.29873	3	G1
7	snp_rs7804806_1wa1_r	rs7804806	AIMS	75595682	0.444	0.349	0.09569	1	G1
7	snp_rs4020771_0w_r	rs4020771	AIMS	78777397	0.649	0.414	0.23587	3	G1
7	snp_rs7783895_0w_f	rs7783895	AncAm	79159240	0.387	0.653	0.26562	3	G2
7	hmrs10258421_f	rs10258421	AIMS	80131842	0.042	0.000	0.04223	1	G1
7	snp_rs7789583_0w_r	rs7789583	AIMS	82965793	0.609	0.894	0.28555	3	G2
7	snp_rs2373124_0w_r	rs2373124	AIMS	85830272	0.111	0.232	0.12108	2	G2
7	snp_rs6949448_0w_f	rs6949448	AIMS	87141814	0.611	0.465	0.14582	2	G1
7	snp_rs6943877_0w_f	rs6943877	AIMS	88591502	0.555	0.669	0.11473	2	G2
7	snp_rs2974129_0w_r	rs2974129	AIMS	91299161	0.345	0.439	0.09405	1	G2
7	snp_rs7810211_0w_f	rs7810211	AIMS	92790829	0.056	0.000	0.05591	1	G1
7	snp_rs1207692_0w_r	rs1207692	AIMS	96578640	0.293	0.489	0.19628	2	G2
7	snp_rs6465748_0w_r	rs6465748	AIMS	98861307	0.085	0.000	0.08525	1	G1
7	snp_rs4730287_0w_f	rs4730287	AIMS	107770624	0.516	0.365	0.15089	2	G1
7	snp_rs7781715_0w_f	rs7781715	AIMS	110713220	0.911	0.994	0.08283	1	G2
7	snp_rs1404228_0w_f	rs1404228	AIMS	113050788	0.199	0.354	0.15511	2	G2
7	snp_rs12705973_0w_f	rs12705973	AIMS	114313199	0.594	0.787	0.19317	2	G2
7	snp_rs6466573_0w_f	rs6466573	AIMS	115949014	0.661	0.531	0.13048	2	G1
7	snp_rs9640779_0w_r	rs9640779	AIMS	117682101	0.335	0.814	0.47909	5	G2
7	snp_rs2896309_0w_f	rs2896309	AIMS	121431954	0.126	0.010	0.11651	2	G1
7	snp_rs1346364_0w_r	rs1346364	AIMS	124930757	0.289	0.136	0.15326	2	G1
7	snp_rs6950264_0w_f	rs6950264	AncAm	126772602	0.444	0.084	0.35998	4	G1
7	snp_rs350655_0w_f	rs350655	AIMS	130713701	0.072	0.034	0.03805	1	G1
7	snp_rs4078096_0w_f	rs4078096	AncAm	131358281	0.620	0.017	0.60238	7	G1
7	hmrs1593306_f	rs1593306	AIMS	131613963	0.013	0.125	0.11265	2	G2
7	snp_rs6952869_0w_r	rs6952869	AIMS	133709787	0.383	0.100	0.28238	3	G1
7	snp_rs11543821_0w_f	rs11543821	AIMS	134834499	0.308	0.489	0.18043	2	G2
7	snp_rs1469179_0w_f	rs1469179	AIMS	136621677	0.504	0.058	0.44642	5	G1
7	snp_rs11763343_0w_f	rs11763343	AIMS	137962186	0.761	0.958	0.19654	2	G2
7	snp_rs2355787_0w_r	rs2355787	AIMS	139089928	0.247	0.198	0.04900	1	G1
7	snp_rs11761774_0w_r	rs11761774	AIMS	141861469	0.865	0.464	0.40025	5	G1
7	snp_rs1973815_0w_f	rs1973815	AIMS	144730377	0.066	0.002	0.06313	1	G1
7	snp_rs12669573_0w_f	rs12669573	AncAm	145193978	0.321	0.550	0.22891	3	G2
7	snp_rs2178768_0w_f	rs2178768	AncAm	147650273	0.374	0.033	0.34117	4	G1
7	snp_rs4571660_0w_f	rs4571660	AIMS	147725461	0.480	0.184	0.29563	3	G1
7	snp_rs6464211_0w_f	rs6464211	AIMS	151873853	0.246	0.384	0.13823	2	G2
7	snp_rs7803356_0w_r	rs7803356	AncAm	153402084	0.443	0.071	0.37187	4	G1
7	hmrs768982_f	rs768982	AIMS	153545621	0.167	0.191	0.02411	1	G2
7	snp_rs9769506_0w_r	rs9769506	AIMS	155099855	0.445	0.375	0.07027	1	G1
7	snp_rs6459830_0w_r	rs6459830	AIMS	157834728	0.333	0.295	0.03859	1	G1
8	snp_rs11784513_0w_f	rs11784513	AIMS	315843	0.856	0.763	0.09273	1	G1
8	snp_rs3860880_0w_r	rs3860880	AIMS	809497	0.412	0.136	0.27604	3	G1
8	snp_rs10108270_0w_r	rs10108270	AIMS	4190793	0.744	0.953	0.20956	3	G2
8	snp_rs12546593_0w_r	rs12546593	AncAm	6677842	0.563	0.387	0.17604	2	G1
8	snp_rs2736340_0w_r	rs2736340	AIMS	11343973	0.360	0.322	0.03748	1	G1
8	snp_rs4367597_0w_f	rs4367597	AIMS	11793529	0.406	0.091	0.31488	4	G1
8	snp_rs11778591_0w_f	rs11778591	AncAm	12720349	0.418	0.837	0.41950	5	G2
8	snp_rs2946513_0w_r	rs2946513	AIMS	12866797	0.439	0.930	0.49081	5	G2
8	snp_rs4416821_0w_r	rs4416821	AIMS	15559730	0.358	0.266	0.09141	1	G1
8	snp_rs1548353_0w_f	rs1548353	AIMS	17166765	0.235	0.428	0.19279	2	G2
8	snp_rs6586906_0w_r	rs6586906	AIMS	20178675	0.216	0.191	0.02469	1	G1
8	snp_rs9325872_0w_f	rs9325872	AncAm	20480271	0.311	0.060	0.25104	3	G1
8	snp_rs4565458_0w_f	rs4565458	AIMS	22154133	0.513	0.713	0.20001	3	G2
8	snp_rs920300_0w_f	rs920300	AIMS	23554565	0.239	0.212	0.02708	1	G1
8	snp_rs12548107_0w_r	rs12548107	AIMS	27057879	0.141	0.062	0.07944	1	G1
8	snp_rs6996714_0w_f	rs6996714	AIMS	28454447	0.138	0.019	0.11932	2	G1
8	snp_rs4352803_0w_f	rs4352803	AIMS	29817551	0.283	0.211	0.07114	1	G1
8	snp_rs13273386_0w_r	rs13273386	AIMS	35645991	0.070	0.000	0.07028	1	G1
8	hmrs6474513_f	rs6474513	AIMS	38804226	0.373	0.760	0.38670	4	G2
8	snp_rs1127379_0w_r	rs1127379	AIMS	41121280	0.546	0.587	0.04139	1	G2
8	snp_rs10448011_0w_f	rs10448011	AncAm	50010896	0.370	0.582	0.21257	3	G2
8	snp_rs1480391_0w_r	rs1480391	AIMS	50108420	0.828	0.611	0.21719	3	G1
8	snp_rs7830163_0w_r	rs7830163	AIMS	53078442	0.247	0.403	0.15678	2	G2
8	snp_rs6993747_0w_f	rs6993747	AIMS	54180915	0.321	0.382	0.06093	1	G2
8	snp_rs997855_0w_f	rs997855	AIMS	56059868	0.359	0.368	0.00883	1	G2
8	snp_rs4316149_0w_r	rs4316149	AIMS	57533247	0.810	0.595	0.21473	3	G1
8	snp_rs1160133_0w_f	rs1160133	AncAm	59305290	0.745	0.130	0.61554	7	G1
8	snp_rs2931309_0w_f	rs2931309	AncAm	62096568	0.483	0.008	0.47497	5	G1
8	snp_rs7820685_0w_f	rs7820685	AIMS	62316409	0.297	0.416	0.11869	2	G2
8	snp_rs2061133_0w_f	rs2061133	AIMS	63830366	0.698	0.881	0.18273	2	G2
8	snp_rs2100347_0w_f	rs2100347	AncAm	64499604	0.525	0.073	0.45177	5	G1
8	snp_rs3110289_0w_r	rs3110289	AIMS	65346936	0.097	0.000	0.09699	1	G1
8	hmrs12546220_r	rs12546220	AncAm	70373728	0.699	0.090	0.60823	7	G1
8	snp_rs2380646_0w_f	rs2380646	AIMS	71358464	0.208	0.303	0.09478	1	G2
8	snp_rs10504595_0w_f	rs10504595	AIMS	76576055	0.105	0.182	0.07696	1	G2
8	snp_rs11784678_0w_r	rs11784678	AIMS	80288217	0.162	0.221	0.05947	1	G2
8	snp_rs7845376_0w_f	rs7845376	AIMS	83222777	0.129	0.535	0.40580	5	G2
8	snp_rs9643769_0w_f	rs9643769	AIMS	84310856	0.452	0.424	0.02871	1	G1
8	snp_rs17623943_0w_f	rs17623943	AIMS	88117846	0.716	0.504	0.21206	3	G1
8	snp_rs10092877_0w_f	rs10092877	AIMS	89823675	0.200	0.193	0.00675	1	G1
8	snp_rs3735912_0w_r	rs3735912	AIMS	93475137	0.051	0.038	0.01295	1	G1
8	snp_rs4304300_0w_f	rs4304300	AIMS	94605374	0.672	0.189	0.48258	5	G1
8	hmrs4734283_f	rs4734283	AIMS	95628814	0.181	0.212	0.03106	1	G2
8	snp_rs2575735_0w_f	rs2575735	AncAm	97534651	0.399	0.858	0.45905	5	G2
8	snp_rs2331465_0w_f	rs2331465	AIMS	98428772	0.678	0.734	0.05601	1	G2
8	snp_rs921313_0w_r	rs921313	AIMS	100824550	0.052	0.118	0.06623	1	G2
8	snp_rs16876241_0w_r	rs16876241	AIMS	108420688	0.113	0.000	0.11265	2	G1
8	snp_rs2980618_0w_r	rs2980618	AIMS	110301998	0.521	0.709	0.18850	2	G2
8	snp_rs6994396_0w_r	rs6994396	AIMS	114811990	0.565	0.144	0.42070	5	G1
8	snp_rs7017679_0w_r	rs7017679	AncAm	115758712	0.744	0.240	0.50381	6	G1
8	snp_rs4876363_0w_r	rs4876363	AIMS	117636329	0.404	0.526	0.12169	2	G2
8	snp_rs4871195_0w_r	rs4871195	AIMS	122498253	0.451	0.699	0.24836	3	G2
8	snp_rs7844723_0w_f	rs7844723	AIMS	122908503	0.431	0.720	0.28879	3	G2
8	hmrs7012891_r	rs7012891	AIMS	126514676	0.513	0.170	0.34342	4	G1
8	snp_rs16901004_0w_r	rs16901004	AncAm	126971002	0.756	0.110	0.64616	7	G1
8	snp_rs7833560_0w_r	rs7833560	AIMS	128072019	0.256	0.657	0.40070	5	G2
8	snp_rs10505517_0w_r	rs10505517	AIMS	129478011	0.092	0.208	0.11664	2	G2
8	snp_rs3829032_0w_f	rs3829032	AIMS	131833036	0.187	0.025	0.16230	2	G1
8	snp_rs10956647_0w_f	rs10956647	AncAm	133202844	0.606	0.127	0.47877	5	G1
8	snp_rs4736413_0w_f	rs4736413	AIMS	133325922	0.456	0.242	0.21426	3	G1
8	hmrs2958830_r	rs2958830	AIMS	134649346	0.137	0.290	0.15219	2	G2
8	snp_rs868421_0w_f	rs868421	AIMS	138599067	0.119	0.058	0.06081	1	G1
9	snp_rs9298675_1wa0_r	rs9298675	AIMS	1252572	0.070	0.010	0.06007	1	G1
9	hmrs4740717_r	rs4740717	AIMS	3183137	0.398	0.431	0.03344	1	G2
9	snp_rs396861_0w_f	rs396861	AIMS	4743626	0.166	0.348	0.18177	2	G2
9	snp_rs7861943_0w_r	rs7861943	AncAm	4836811	0.473	0.782	0.30913	4	G2
9	snp_rs10119431_0w_f	rs10119431	AncAm	7759331	0.468	0.716	0.24823	3	G2
9	snp_rs7043322_0w_f	rs7043322	AIMS	8241845	0.011	0.056	0.04497	1	G2
9	snp_rs1500318_0w_r	rs1500318	AIMS	8361445	0.091	0.067	0.02365	1	G1
9	snp_rs1333111_0w_r	rs1333111	AncAm	9416962	0.485	0.086	0.39855	4	G1
9	snp_rs1408125_0w_r	rs1408125	AIMS	9737201	0.710	1.000	0.28993	3	G2
9	snp_rs16929332_0w_r	rs16929332	AncAm	12671693	0.440	0.387	0.05301	1	G1
9	snp_rs2382371_0w_r	rs2382371	AIMS	12780295	0.105	0.102	0.00280	1	G1
9	snp_rs566820_0w_r	rs566820	AIMS	15192720	0.141	0.000	0.14053	2	G1
9	snp_rs10962589_0w_r	rs10962589	AIMS	16783380	0.644	0.462	0.18249	2	G1
9	snp_rs2840790_0w_f	rs2840790	AIMS	17969519	0.442	0.000	0.44221	5	G1
9	snp_rs4468024_0w_f	rs4468024	AIMS	19294073	0.123	0.447	0.32442	4	G2
9	snp_rs9792640_0w_f	rs9792640	AncAm	20035279	0.414	0.084	0.32933	4	G1
9	snp_rs7037813_0w_35AC_r	rs7037813	AIMS	20353227	0.211	0.749	0.53795	6	G2
9	snp_rs10511715_0w_f	rs10511715	AIMS	23079238	0.597	0.634	0.03758	1	G2
9	snp_rs4977638_0w_f	rs4977638	AncAm	24055823	0.605	1.000	0.39457	4	G2
9	snp_rs10511745_0w_f	rs10511745	AIMS	24786754	0.820	0.812	0.00858	1	G1
9	snp_rs1231375_0w_f	rs1231375	AncAm	25755952	0.281	0.883	0.60215	7	G2
9	snp_rs10812520_0w_r	rs10812520	AIMS	27047246	0.877	0.918	0.04104	1	G2
9	snp_rs1901565_0w_r	rs1901565	AIMS	28055900	0.509	0.202	0.30669	4	G1
9	snp_rs2888998_0w_r	rs2888998	AIMS	30979640	0.410	0.482	0.07225	1	G2
9	snp_rs867469_0w_f	rs867469	AIMS	32383708	0.560	0.382	0.17841	2	G1
9	hmrs7853023_f	rs7853023	AIMS	38772575	0.854	0.728	0.12603	2	G1
9	snp_rs1180109_0w_r	rs1180109	AIMS	72885267	0.827	0.870	0.04303	1	G2
9	snp_rs2147241_0w_f	rs2147241	AIMS	74602933	0.271	0.742	0.47074	5	G2
9	snp_rs2130118_0w_r	rs2130118	AIMS	76343289	0.376	0.285	0.09085	1	G1
9	snp_rs2094583_0w_f	rs2094583	AIMS	78263400	0.609	0.430	0.17873	2	G1
9	snp_rs17064021_0w_r	rs17064021	AIMS	80616526	0.000	0.054	0.05356	1	G2
9	snp_rs7851008_0w_r	rs7851008	AncAm	82596501	0.289	1.000	0.71143	8	G2
9	snp_rs11139177_0w_f	rs11139177	AncAm	83986875	0.520	0.000	0.52024	6	G1
9	snp_rs2251760_0w_r	rs2251760	AncAm	86811147	0.735	0.822	0.08665	1	G2
9	hmrs10746763_f	rs10746763	AIMS	88131557	0.373	0.773	0.40026	5	G2
9	snp_rs4877464_0w_r	rs4877464	AIMS	91229667	0.733	0.528	0.20458	3	G1
9	snp_rs7042102_0w_f	rs7042102	AIMS	94763790	0.323	0.275	0.04790	1	G1
9	snp_rs2255840_0w_f	rs2255840	AIMS	96493773	0.222	0.268	0.04663	1	G2
9	snp_rs10759835_0w_35AC_r	rs10759835	AIMS	98546523	0.214	0.317	0.10379	2	G2
9	snp_rs884886_0w_r	rs884886	AIMS	101318075	0.678	0.521	0.15659	2	G1
9	snp_rs7039994_0w_f	rs7039994	AIMS	103051205	0.084	0.000	0.08366	1	G1
9	snp_rs10989064_0w_r	rs10989064	AncAm	103111965	0.397	0.000	0.39724	4	G1
9	snp_rs320201_0w_f	rs320201	AIMS	104941304	0.355	0.219	0.13658	2	G1
9	snp_rs10820100_0w_f	rs10820100	AncAm	104989394	0.121	0.793	0.67141	7	G2
9	hmrs2771036_f	rs2771036	AIMS	108205262	0.167	0.000	0.16688	2	G1
9	snp_rs7867544_0w_f	rs7867544	AIMS	110013980	0.070	0.719	0.64859	7	G2
9	snp_rs7047831_0w_f	rs7047831	AncAm	110688551	0.409	1.000	0.59054	6	G2
9	snp_rs10739277_0w_r	rs10739277	AIMS	112051958	0.022	0.030	0.00842	1	G2
9	snp_rs7875749_0w_r	rs7875749	AncAm	114387135	0.403	0.135	0.26765	3	G1
9	snp_rs7860625_0w_f	rs7860625	AIMS	116065087	0.114	0.000	0.11350	2	G1
9	snp_rs6478156_0w_r	rs6478156	AncAm	118024692	0.508	0.935	0.42659	5	G2
9	snp_rs2809393_0w_f	rs2809393	AncAm	120891637	0.463	0.186	0.27696	3	G1
9	snp_rs12340264_0w_f	rs12340264	AIMS	124109921	0.815	0.446	0.36968	4	G1
9	snp_rs2778635_0w_r	rs2778635	AIMS	125229799	0.572	0.276	0.29638	3	G1
9	snp_rs1476859_0w_f	rs1476859	AncAm	125391127	0.328	0.280	0.04750	1	G1
9	snp_rs10818918_1wa0_r	rs10818918	AncAm	126860510	0.562	0.000	0.56192	6	G1
9	hmrs871391_f	rs871391	AIMS	128420381	0.780	0.329	0.45075	5	G1
9	snp_rs516104_0w_r	rs516104	AIMS	129580187	0.735	0.555	0.18002	2	G1
9	snp_rs2417192_0w_f	rs2417192	AIMS	132771351	0.125	0.268	0.14310	2	G2
10	snp_rs1901632_0w_r	rs1901632	AIMS	4813211	0.207	0.197	0.01027	1	G1
10	snp_rs2669130_0w_f	rs2669130	AncAm	5982577	0.531	0.008	0.52326	6	G1
10	snp_rs7921493_0w_f	rs7921493	AIMS	7339064	0.078	0.208	0.12933	2	G2
10	snp_rs11255712_0w_f	rs11255712	AIMS	8511627	0.472	0.616	0.14390	2	G2
10	snp_rs2815611_0w_f	rs2815611	AIMS	12501398	0.202	0.103	0.09846	1	G1
10	snp_rs1409392_0w_r	rs1409392	AIMS	14621153	0.612	0.713	0.10163	2	G2
10	snp_rs7903263_0w_f	rs7903263	AncAm	14698952	0.539	0.733	0.19392	2	G2
10	snp_rs3012478_0w_r	rs3012478	AIMS	17035394	0.781	0.552	0.22862	3	G1
10	snp_rs993864_0w_r	rs993864	AIMS	18539795	0.193	0.147	0.04669	1	G1
10	snp_rs7083165_0w_f	rs7083165	AncAm	20516587	0.171	0.887	0.71597	8	G2
10	snp_rs2666771_0w_r	rs2666771	AIMS	22148230	0.652	0.608	0.04484	1	G1
10	snp_rs1414443_1wa0_r	rs1414443	AIMS	24115017	0.225	0.096	0.12881	2	G1
10	snp_rs2067701_0w_f	rs2067701	AncAm	24326623	0.718	0.947	0.22945	3	G2
10	snp_rs2484873_0w_r	rs2484873	AncAm	24634956	0.249	0.646	0.39621	4	G2
10	hmrs9416795_f	rs9416795	AIMS	28709550	0.105	0.025	0.08021	1	G1
10	snp_rs4747667_0w_r	rs4747667	AIMS	29836410	0.517	0.363	0.15378	2	G1
10	snp_rs10508773_0w_r	rs10508773	AIMS	32575444	0.206	0.252	0.04589	1	G2
10	snp_rs2605506_0w_r	rs2605506	AIMS	33798746	0.279	0.663	0.38372	4	G2
10	hmrs7906685_f	rs7906685	AIMS	36379503	0.231	0.566	0.33454	4	G2
10	snp_rs12243792_0w_r	rs12243792	AIMS	38081716	0.054	0.378	0.32356	4	G2
10	snp_rs4341471_0w_r	rs4341471	AIMS	48521126	0.243	0.232	0.01048	1	G1
10	snp_rs3793791_0w_r	rs3793791	AIMS	50841704	0.256	0.650	0.39315	4	G2
10	snp_rs2795918_0w_r	rs2795918	Both	56177015	0.540	0.078	0.46153	5	G1
10	snp_rs3862864_0w_f	rs3862864	AIMS	59912870	0.045	0.015	0.02998	1	G1
10	snp_rs1684895_0w_f	rs1684895	AIMS	61726870	0.365	0.053	0.31139	4	G1
10	snp_rs17457687_0w_r	rs17457687	AIMS	69911574	0.000	0.456	0.45567	5	G2
10	hmrs499437_r	rs499437	AIMS	78993943	0.555	0.745	0.19013	2	G2
10	snp_rs7915399_0w_f	rs7915399	AIMS	83996992	0.019	0.195	0.17559	2	G2
10	snp_rs1857459_0w_r	rs1857459	AIMS	85684595	0.298	0.451	0.15214	2	G2
10	hmrs10785942_r	rs10785942	AIMS	91902898	0.235	0.453	0.21848	3	G2
10	snp_rs2245251_0w_r	rs2245251	AIMS	93060457	0.523	0.881	0.35789	4	G2
10	snp_rs10882029_0w_f	rs10882029	AncAm	93955851	0.706	0.368	0.33757	4	G1
10	snp_rs10882147_0w_f	rs10882147	AncAm	94877718	0.442	0.874	0.43260	5	G2
10	snp_rs701873_0w_r	rs701873	AIMS	95190568	0.569	0.744	0.17430	2	G2
10	snp_rs1023331_0w_f	rs1023331	AIMS	97766537	0.590	0.860	0.27058	3	G2
10	hmrs563654_f	rs563654	AIMS	98478449	0.649	0.354	0.29512	3	G1
10	snp_rs1060445_0w_f	rs1060445	AIMS	100218766	0.321	0.499	0.17780	2	G2
10	snp_rs2475435_0w_r	rs2475435	AIMS	102924363	0.147	0.146	0.00092	1	G1
10	snp_rs7071247_1wa0_r	rs7071247	AIMS	105257786	0.206	0.211	0.00530	1	G2
10	snp_rs10509826_0w_f	rs10509826	AIMS	108804840	0.348	0.128	0.22076	3	G1
10	snp_rs1936488_0w_r	rs1936488	AIMS	109909743	0.775	0.743	0.03222	1	G1
10	snp_rs17127304_0w_f	rs17127304	AIMS	112134500	0.887	1.000	0.11266	2	G2
10	snp_rs1325172_0w_r	rs1325172	AIMS	114358555	0.086	0.043	0.04318	1	G1
10	snp_rs4261225_0w_r	rs4261225	AncAm	115289375	0.582	0.099	0.48304	5	G1
10	hmrs17094083_r	rs17094083	AIMS	117860851	0.325	0.342	0.01679	1	G2
10	snp_rs7087634_0w_f	rs7087634	AncAm	118171016	0.416	0.730	0.31339	4	G2
10	hmrs7906403_f	rs7906403	AIMS	125109478	0.155	0.000	0.15476	2	G1
10	snp_rs12243508_0w_r	rs12243508	AIMS	127997728	0.795	0.686	0.10949	2	G1
10	snp_rs10829838_0w_f	rs10829838	AIMS	129158853	0.102	0.382	0.27984	3	G2
10	snp_rs982836_0w_f	rs982836	AIMS	130476845	0.294	0.657	0.36275	4	G2
10	snp_rs11017252_0w_f	rs11017252	AncAm	132231596	0.171	0.621	0.45021	5	G2
10	hmrs2397769_f	rs2397769	AIMS	133120700	0.247	0.476	0.22898	3	G2
11	snp_rs2570580_0w_f	rs2570580	AIMS	4938352	0.092	0.022	0.07019	1	G1
11	snp_rs1506981_0w_r	rs1506981	AIMS	6779071	0.270	0.175	0.09565	1	G1
11	snp_rs1105984_0w_f	rs1105984	AIMS	8016730	0.247	0.172	0.07485	1	G1
11	snp_rs2872220_0w_f	rs2872220	AIMS	9615459	0.086	0.070	0.01544	1	G1
11	snp_rs1378357_0w_f	rs1378357	AIMS	11802361	0.122	0.056	0.06554	1	G1
11	snp_rs10741584_0w_f	rs10741584	AIMS	12383869	0.099	0.008	0.09148	1	G1
11	snp_rs745361_0w_r	rs745361	AIMS	12946203	0.069	0.018	0.05137	1	G1
11	snp_rs16930498_0w_f	rs16930498	AIMS	14757095	0.062	0.103	0.04082	1	G2
11	snp_rs7129202_0w_r	rs7129202	AncAm	19830495	0.431	0.685	0.25452	3	G2
11	snp_rs7931276_1wa1_f	rs7931276	AIMS	21649811	0.104	0.236	0.13119	2	G2
11	snp_rs7118075_0w_f	rs7118075	AncAm	22341713	0.488	0.000	0.48755	5	G1
11	snp_rs11027552_0w_f	rs11027552	AIMS	23859991	0.689	0.495	0.19431	2	G1
11	snp_rs1477569_1wa0_f	rs1477569	AIMS	25230780	0.799	1.000	0.20070	3	G2
11	snp_rs7949057_0w_r	rs7949057	AIMS	28255053	0.706	1.000	0.29359	3	G2
11	snp_rs2993051_0w_r	rs2993051	Both	29994465	0.438	0.894	0.45569	5	G2
11	snp_rs533448_0w_f	rs533448	AIMS	30012458	0.232	0.032	0.20017	3	G1
11	snp_rs4593985_0w_f	rs4593985	AIMS	35403124	0.612	0.473	0.13931	2	G1
11	snp_rs1030774_1wa0_f	rs1030774	AIMS	38497601	0.772	0.713	0.05846	1	G1
11	snp_rs4755538_0w_f	rs4755538	AIMS	40047754	0.547	0.370	0.17697	2	G1
11	snp_rs10501292_0w_r	rs10501292	AIMS	42996387	0.128	0.000	0.12778	2	G1
11	snp_rs2863174_0w_f	rs2863174	AncAm	45264277	0.222	0.679	0.45617	5	G2
11	hmrs2863720_r	rs2863720	AIMS	46235626	0.427	0.000	0.42692	5	G1
11	snp_rs1603756_0w_f	rs1603756	AIMS	51314773	0.493	0.266	0.22748	3	G1
11	snp_rs9666086_0w_f	rs9666086	AIMS	55703650	0.057	0.000	0.05657	1	G1
11	snp_rs2218868_0w_f	rs2218868	AIMS	57925977	0.323	0.435	0.11179	2	G2
11	snp_rs2237997_0w_f	rs2237997	AIMS	60772090	0.366	0.000	0.36557	4	G1
11	snp_rs12804561_0w_r	rs12804561	AIMS	62916577	0.428	0.071	0.35777	4	G1
11	snp_rs2904979_0w_f	rs2904979	AIMS	65000719	0.083	0.134	0.05181	1	G2
11	snp_rs12422149_0w_f	rs12422149	AncAm	74883577	0.713	0.294	0.41836	5	G1
11	snp_rs2508241_0w_r	rs2508241	AIMS	78882172	0.297	0.095	0.20228	3	G1
11	snp_rs7342241_0w_r	rs7342241	AIMS	80707611	0.218	0.040	0.17859	2	G1
11	snp_rs11607377_0w_r	rs11607377	AIMS	82818226	0.209	0.294	0.08460	1	G2
11	snp_rs568789_0w_r	rs568789	AIMS	84146142	0.286	0.137	0.14887	2	G1
11	snp_rs11823687_0w_r	rs11823687	AIMS	84577299	0.014	0.000	0.01398	1	G1
11	snp_rs4753486_0w_r	rs4753486	AIMS	88258689	0.550	0.324	0.22590	3	G1
11	snp_rs1042602_0w_f	rs1042602	AIMS	88911696	0.352	0.052	0.29966	3	G1
11	snp_rs4268468_0w_f	rs4268468	AncAm	89886284	0.688	0.133	0.55563	6	G1
11	snp_rs1370018_0w_f	rs1370018	AIMS	91016008	0.914	0.886	0.02839	1	G1
11	snp_rs956267_0w_f	rs956267	AIMS	92742186	0.230	0.236	0.00675	1	G2
11	snp_rs2248020_0w_f	rs2248020	AncAm	93259965	0.728	0.088	0.63963	7	G1
11	snp_rs526108_0w_r	rs526108	AIMS	95154173	0.107	0.030	0.07676	1	G1
11	snp_rs2406073_0w_f	rs2406073	AIMS	98379690	0.592	0.471	0.12066	2	G1
11	snp_rs2897642_0w_r	rs2897642	AIMS	99812735	0.348	0.765	0.41729	5	G2
11	snp_rs551632_0w_f	rs551632	AncAm	100856955	0.703	0.035	0.66816	7	G1
11	snp_rs7121108_0w_f	rs7121108	AIMS	101452331	0.126	0.000	0.12627	2	G1
11	snp_rs488753_0w_r	rs488753	AIMS	103976293	0.312	0.000	0.31167	4	G1
11	snp_rs10502069_0w_f	rs10502069	AIMS	106316611	0.151	0.002	0.14846	2	G1
11	snp_rs1800889_0w_f	rs1800889	AIMS	108163487	0.090	0.049	0.04071	1	G1
11	snp_rs317519_0w_r	rs317519	AIMS	110890394	0.769	0.560	0.20842	3	G1
11	snp_rs1447460_0w_r	rs1447460	AIMS	112736597	0.819	0.911	0.09180	1	G2
11	snp_rs220859_0w_r	rs220859	AncAm	115312071	0.499	0.972	0.47305	5	G2
11	snp_rs7131355_0w_r	rs7131355	AIMS	116077370	0.237	0.329	0.09268	1	G2
11	snp_rs3802871_0w_f	rs3802871	AIMS	117776648	0.516	0.579	0.06290	1	G2
11	snp_rs643788_0w_f	rs643788	AIMS	118967758	0.591	0.964	0.37361	4	G2
11	snp_rs948028_0w_r	rs948028	AIMS	120644447	0.180	0.356	0.17621	2	G2
11	snp_rs11605969_1wa1_f	rs11605969	AIMS	121430872	0.858	1.000	0.14237	2	G2
11	snp_rs10893032_0w_f	rs10893032	AIMS	123246154	0.226	0.096	0.12987	2	G1
11	snp_rs1148085_0w_r	rs1148085	AIMS	123527069	0.305	0.268	0.03700	1	G1
11	snp_rs4936969_0w_f	rs4936969	AIMS	124890396	0.791	0.680	0.11139	2	G1
11	snp_rs649142_0w_r	rs649142	AIMS	126043464	0.543	1.000	0.45715	5	G2
11	hmrs485645_f	rs485645	Both	126432979	0.416	0.989	0.57305	6	G2
11	snp_rs7949452_0w_f	rs7949452	AIMS	127294959	0.578	0.963	0.38518	4	G2
11	snp_rs4372467_0w_r	rs4372467	AIMS	128382804	0.313	0.790	0.47708	5	G2
11	snp_rs373665_0w_f	rs373665	AIMS	131406707	0.172	0.273	0.10027	2	G2
11	snp_rs1901916_0w_f	rs1901916	AIMS	132964299	0.408	0.461	0.05299	1	G2
12	snp_rs527118_0w_f	rs527118	AncAm	371410	0.494	0.230	0.26381	3	G1
12	hmrs735295_f	rs735295	AIMS	547683	0.432	0.614	0.18195	2	G2
12	snp_rs10774171_0w_r	rs10774171	AncAm	3868309	0.347	0.864	0.51730	6	G2
12	snp_rs534665_0w_r	rs534665	AIMS	5321472	0.669	0.553	0.11646	2	G1
12	snp_rs7976721_0w_r	rs7976721	AIMS	7933635	0.027	0.010	0.01742	1	G1
12	snp_rs2416791_0w_f	rs2416791	AIMS	11701488	0.871	0.510	0.36096	4	G1
12	snp_rs1158541_0w_f	rs1158541	AIMS	13846673	0.605	0.558	0.04703	1	G1
12	snp_rs4477487_0w_f	rs4477487	AIMS	17892560	0.148	0.119	0.02878	1	G1
12	snp_rs10770437_0w_f	rs10770437	AIMS	19299390	0.050	0.094	0.04464	1	G2
12	snp_rs10770658_0w_f	rs10770658	AncAm	20586821	0.455	0.999	0.54345	6	G2
12	snp_rs1479446_0w_r	rs1479446	AIMS	23801976	0.306	0.012	0.29414	3	G1
12	snp_rs699039_0w_f	rs699039	AIMS	25164331	0.749	0.974	0.22493	3	G2
12	snp_rs7979400_0w_f	rs7979400	AncAm	26943416	0.317	0.932	0.61487	7	G2
12	snp_rs4265668_0w_f	rs4265668	AIMS	27338185	0.355	0.012	0.34245	4	G1
12	snp_rs11613738_0w_r	rs11613738	AIMS	30886435	0.104	0.004	0.09967	1	G1
12	snp_rs12370505_0w_f	rs12370505	AIMS	33089955	0.186	0.300	0.11375	2	G2
12	snp_rs1497050_0w_r	rs1497050	AIMS	40307598	0.501	0.707	0.20571	3	G2
12	snp_rs2408141_0w_f	rs2408141	AIMS	45503563	0.620	0.415	0.20570	3	G1
12	snp_rs10783299_0w_f	rs10783299	AIMS	49390677	0.382	0.308	0.07363	1	G1
12	snp_rs589619_0w_r	rs589619	AncAm	53036090	0.533	0.959	0.42601	5	G2
12	snp_rs4374022_0w_f	rs4374022	AIMS	55373780	0.398	0.556	0.15819	2	G2
12	snp_rs10877071_0w_f	rs10877071	AIMS	58498926	0.651	0.875	0.22393	3	G2
12	snp_rs1391129_0w_f	rs1391129	AIMS	59992011	0.281	0.567	0.28640	3	G2
12	snp_rs795342_0w_r	rs795342	AIMS	61464968	0.276	0.104	0.17248	2	G1
12	snp_rs2088170_0w_r	rs2088170	AIMS	66782412	0.572	0.703	0.13041	2	G2
12	snp_rs10506555_0w_f	rs10506555	AIMS	67909350	0.182	0.133	0.04903	1	G1
12	snp_rs2860493_0w_f	rs2860493	AIMS	68922850	0.281	0.346	0.06495	1	G2
12	hmrs10444584_f	rs10444584	AIMS	71539646	0.537	0.861	0.32398	4	G2
12	snp_rs10879274_0w_r	rs10879274	AncAm	71633978	0.869	0.148	0.72093	8	G1
12	snp_rs954147_0w_r	rs954147	AIMS	72903301	0.793	0.634	0.15989	2	G1
12	snp_rs11180619_0w_f	rs11180619	AIMS	76048838	0.359	0.465	0.10561	2	G2
12	hmrs6582298_f	rs6582298	AncAm	76114872	0.554	0.975	0.42154	5	G2
12	snp_rs4842309_0w_r	rs4842309	AIMS	79403715	0.801	0.691	0.11038	2	G1
12	snp_rs7304562_0w_r	rs7304562	AIMS	80444611	0.039	0.513	0.47345	5	G2
12	snp_rs17709807_0w_r	rs17709807	AIMS	82575933	0.424	0.308	0.11554	2	G1
12	snp_rs10466960_0w_f	rs10466960	AIMS	83249247	0.033	0.061	0.02787	1	G2
12	snp_rs10161479_0w_f	rs10161479	AIMS	85356224	0.531	0.587	0.05563	1	G2
12	snp_rs10506937_0w_r	rs10506937	AIMS	87301493	0.449	0.674	0.22536	3	G2
12	snp_rs2723891_0w_f	rs2723891	AIMS	90958258	0.264	0.481	0.21727	3	G2
12	snp_rs11106213_0w_f	rs11106213	AIMS	92008193	0.152	0.037	0.11488	2	G1
12	snp_rs11107018_0w_r	rs11107018	AIMS	93813902	0.051	0.189	0.13843	2	G2
12	snp_rs7307510_0w_r	rs7307510	AIMS	96237570	0.180	0.000	0.18007	2	G1
12	snp_rs12231308_0w_r	rs12231308	AIMS	97517174	0.555	0.571	0.01599	1	G2
12	snp_rs2373201_0w_r	rs2373201	AIMS	100332815	0.093	0.040	0.05304	1	G1
12	snp_rs10860548_0w_f	rs10860548	AncAm	100380198	0.471	1.000	0.52879	6	G2
12	snp_rs7300149_0w_f	rs7300149	AIMS	101944579	0.139	0.000	0.13930	2	G1
12	snp_rs7312155_0w_r	rs7312155	AIMS	107210453	0.084	0.000	0.08394	1	G1
12	snp_rs2228315_0w_f	rs2228315	AIMS	109017898	0.088	0.087	0.00092	1	G1
12	snp_rs991817_0w_f	rs991817	AIMS	111410537	0.192	0.174	0.01793	1	G1
12	snp_rs16944514_0w_f	rs16944514	AncAm	115041895	0.572	0.356	0.21606	3	G1
12	snp_rs1955105_0w_r	rs1955105	AIMS	115344782	0.457	0.403	0.05468	1	G1
12	snp_rs1463630_0w_r	rs1463630	AncAm	116060432	0.536	0.291	0.24535	3	G1
12	snp_rs4767387_1wa0_f	rs4767387	AIMS	116634170	0.568	0.412	0.15571	2	G1
12	snp_rs2650170_1wa0_f	rs2650170	AIMS	118021755	0.077	0.191	0.11347	2	G2
12	snp_rs1503767_0w_f	rs1503767	AIMS	118889488	0.320	0.473	0.15283	2	G2
12	snp_rs2698081_0w_f	rs2698081	AncAm	119294242	0.766	0.392	0.37390	4	G1
12	snp_rs1405050_0w_f	rs1405050	AIMS	119443713	0.467	0.000	0.46726	5	G1
12	hmrs427244_r	rs427244	AIMS	124605287	0.166	0.000	0.16626	2	G1
12	snp_rs838905_1wa0_f	rs838905	AncAm	125291006	0.174	0.585	0.41051	5	G2
12	hmrs10744271_f	rs10744271	AIMS	127064417	0.542	0.059	0.48285	5	G1
12	snp_rs11060202_0w_f	rs11060202	AncAm	129693530	0.458	0.154	0.30370	4	G1
12	snp_rs11061330_0w_r	rs11061330	AncAm	131594563	0.593	0.328	0.26466	3	G1
13	snp_rs377318_0w_f	rs377318	AIMS	22886101	0.391	0.560	0.16932	2	G2
13	snp_rs9551080_0w_f	rs9551080	AncAm	24777773	0.533	0.035	0.49823	5	G1
13	snp_rs2863937_0w_r	rs2863937	AIMS	25349710	0.737	0.767	0.02981	1	G2
13	snp_rs7990216_0w_r	rs7990216	AncAm	25900838	0.392	0.731	0.33971	4	G2
13	hmrs12430350_f	rs12430350	AncAm	26755384	0.573	0.000	0.57273	6	G1
13	snp_rs7983897_0w_f	rs7983897	AIMS	28441031	0.156	0.158	0.00182	1	G2
13	snp_rs9805284_0w_f	rs9805284	AIMS	31824650	0.470	0.222	0.24738	3	G1
13	snp_rs878682_0w_r	rs878682	AIMS	33837594	0.048	0.015	0.03289	1	G1
13	snp_rs12583395_unw_f	rs12583395	AIMS	35219778	0.070	0.000	0.06958	1	G1
13	snp_rs7322307_0w_f	rs7322307	AIMS	37350679	0.448	0.668	0.22050	3	G2
13	snp_rs9315923_0w_f	rs9315923	AncAm	43070894	0.320	0.027	0.29263	3	G1
13	snp_rs7323018_0w_r	rs7323018	AIMS	44615039	0.404	0.178	0.22542	3	G1
13	snp_rs2985961_0w_f	rs2985961	AIMS	46071980	0.471	0.456	0.01454	1	G1
13	snp_rs17289394_0w_r	rs17289394	AIMS	47473220	0.650	0.341	0.30887	4	G1
13	snp_rs1000603_0w_f	rs1000603	AIMS	49347268	0.028	0.000	0.02813	1	G1
13	snp_rs9563069_0w_r	rs9563069	AncAm	52303139	0.529	0.078	0.45149	5	G1
13	snp_rs9536454_0w_f	rs9536454	AIMS	54114469	0.070	0.000	0.07001	1	G1
13	snp_rs9527650_0w_r	rs9527650	Both	57962413	0.320	0.033	0.28758	3	G1
13	snp_rs7339413_0w_f	rs7339413	AIMS	62125220	0.569	0.812	0.24339	3	G2
13	snp_rs13379032_0w_f	rs13379032	AIMS	65460917	0.211	0.396	0.18496	2	G2
13	snp_rs17086822_0w_f	rs17086822	AIMS	70925820	0.837	0.757	0.07958	1	G1
13	snp_rs7336486_0w_f	rs7336486	AncAm	72438081	0.425	0.016	0.40902	5	G1
13	snp_rs9543281_0w_f	rs9543281	AncAm	73803901	0.317	0.022	0.29597	3	G1
13	snp_rs9600179_0w_f	rs9600179	AncAm	74440000	0.344	0.040	0.30471	4	G1
13	snp_rs4885346_0w_f	rs4885346	AIMS	76323485	0.721	0.152	0.56856	6	G1
13	snp_rs9574199_0w_f	rs9574199	AncAm	78808914	0.497	0.931	0.43356	5	G2
13	hmrs8001641_r	rs8001641	AIMS	80692811	0.629	0.854	0.22517	3	G2
13	snp_rs12323203_0w_f	rs12323203	AIMS	82087781	0.119	0.040	0.07926	1	G1
13	snp_rs1999513_0w_f	rs1999513	AIMS	84067742	0.222	0.295	0.07324	1	G2
13	snp_rs5009567_0w_f	rs5009567	AncAm	85394807	0.444	0.886	0.44183	5	G2
13	snp_rs7327483_0w_f	rs7327483	AncAm	85722171	0.473	0.057	0.41552	5	G1
13	snp_rs9517465_0w_f	rs9517465	AIMS	88170033	0.382	0.332	0.05024	1	G1
13	snp_rs996292_0w_f	rs996292	AIMS	90184410	0.192	0.579	0.38724	4	G2
13	snp_rs828246_0w_f	rs828246	AIMS	91346989	0.393	0.142	0.25146	3	G1
13	snp_rs10083274_0w_f	rs10083274	AIMS	93112987	0.438	0.477	0.03998	1	G2
13	snp_rs2762111_0w_f	rs2762111	AncAm	94051114	0.572	0.096	0.47633	5	G1
13	hmrs1678386_f	rs1678386	AIMS	95849176	0.535	0.550	0.01469	1	G2
13	snp_rs9556553_0w_f	rs9556553	AIMS	96956093	0.066	0.073	0.00700	1	G2
13	snp_rs2297319_0w_f	rs2297319	AIMS	99361959	0.798	0.684	0.11383	2	G1
13	snp_rs9554742_0w_f	rs9554742	AncAm	101596542	0.539	0.439	0.10038	2	G1
13	snp_rs4429163_0w_r	rs4429163	AIMS	103152549	0.933	0.773	0.16026	2	G1
13	snp_rs951095_1wa1_r	rs951095	AIMS	105478644	0.728	0.189	0.53883	6	G1
13	snp_rs4255644_0w_r	rs4255644	AIMS	111089573	0.181	0.000	0.18072	2	G1
13	snp_rs9522149_0w_f	rs9522149	AIMS	111827167	0.618	0.040	0.57759	6	G1
14	snp_rs943889_0w_r	rs943889	AIMS	23169536	0.139	0.116	0.02260	1	G1
14	snp_rs2332505_0w_f	rs2332505	AIMS	25973402	0.676	0.354	0.32173	4	G1
14	snp_rs6574512_0w_r	rs6574512	AncAm	26077514	0.627	0.182	0.44502	5	G1
14	snp_rs7151991_0w_r	rs7151991	Both	32635572	0.625	0.099	0.52596	6	G1
14	snp_rs10872871_0w_r	rs10872871	AncAm	33386759	0.389	0.670	0.28120	3	G2
14	hmrs7152286_f	rs7152286	AIMS	34276484	0.707	0.403	0.30382	4	G1
14	snp_rs723204_0w_f	rs723204	AIMS	36371632	0.785	0.888	0.10267	2	G2
14	snp_rs6571767_0w_r	rs6571767	AIMS	37400841	0.346	0.420	0.07321	1	G2
14	snp_rs2038281_0w_r	rs2038281	AIMS	39915197	0.339	0.166	0.17297	2	G1
14	snp_rs1475807_unw_f	rs1475807	AIMS	41023693	0.244	0.307	0.06300	1	G2
14	snp_rs11846415_0w_r	rs11846415	AIMS	42408943	0.382	0.513	0.13023	2	G2
14	snp_rs10498365_0w_r	rs10498365	AncAm	42700527	0.272	0.121	0.15130	2	G1
14	snp_rs2009767_0w_f	rs2009767	AIMS	43980703	0.134	0.122	0.01252	1	G1
14	snp_rs11845717_0w_f	rs11845717	AncAm	44622317	0.479	0.849	0.37062	4	G2
14	snp_rs7142055_0w_f	rs7142055	AIMS	45987005	0.531	0.356	0.17448	2	G1
14	snp_rs4898706_0w_f	rs4898706	AIMS	52135356	0.595	0.369	0.22594	3	G1
14	snp_rs1950736_0w_r	rs1950736	AncAm	52600254	0.360	0.766	0.40611	5	G2
14	snp_rs1954333_0w_r	rs1954333	AIMS	53912834	0.243	0.361	0.11815	2	G2
14	snp_rs169748_0w_f	rs169748	AncAm	54186083	0.503	0.181	0.32279	4	G1
14	snp_rs1957358_0w_f	rs1957358	AncAm	55222475	0.350	0.005	0.34467	4	G1
14	snp_rs1188184_0w_r	rs1188184	AncAm	56442412	0.446	0.000	0.44575	5	G1
14	snp_rs2622324_0w_r	rs2622324	AIMS	56530093	0.191	0.011	0.17945	2	G1
14	snp_rs17719756_0w_f	rs17719756	AncAm	57454439	0.509	0.000	0.50887	6	G1
14	snp_rs1950993_0w_f	rs1950993	AIMS	58238687	0.409	0.234	0.17507	2	G1
14	snp_rs10130183_0w_r	rs10130183	AIMS	58698338	0.241	0.544	0.30241	4	G2
14	snp_rs8660_0w_r	rs8660	AIMS	59939727	0.513	0.598	0.08506	1	G2
14	snp_rs7492698_0w_r	rs7492698	AIMS	62740092	0.335	0.757	0.42170	5	G2
14	snp_rs10140978_0w_f	rs10140978	AIMS	64528071	0.044	0.052	0.00817	1	G2
14	snp_rs3825725_0w_f	rs3825725	AIMS	68035879	0.626	0.713	0.08655	1	G2
14	snp_rs886599_0w_r	rs886599	AIMS	71174444	0.635	0.761	0.12576	2	G2
14	snp_rs2543383_0w_f	rs2543383	AIMS	76626703	0.854	0.933	0.07904	1	G2
14	snp_rs12881440_0w_f	rs12881440	AIMS	78472730	0.491	0.577	0.08554	1	G2
14	snp_rs7142344_0w_r	rs7142344	AIMS	79250144	0.000	0.151	0.15115	2	G2
14	snp_rs1531631_0w_r	rs1531631	AIMS	79624492	0.244	0.059	0.18430	2	G1
14	snp_rs8003492_0w_r	rs8003492	AIMS	81085352	0.540	0.760	0.22037	3	G2
14	snp_rs799099_0w_r	rs799099	AIMS	82717397	0.192	0.127	0.06506	1	G1
14	snp_rs1289248_0w_r	rs1289248	AncAm	87028855	0.348	0.020	0.32798	4	G1
14	snp_rs1769463_0w_f	rs1769463	AIMS	87512064	0.244	0.351	0.10727	2	G2
14	snp_rs17776811_0w_r	rs17776811	AncAm	90066451	0.339	0.117	0.22266	3	G1
14	snp_rs17126387_0w_f	rs17126387	AIMS	90301314	0.125	0.009	0.11570	2	G1
14	snp_rs3825663_1wa0_r	rs3825663	AIMS	90429749	0.157	0.036	0.12112	2	G1
14	snp_rs12436076_0w_f	rs12436076	AIMS	91488751	0.235	0.000	0.23485	3	G1
14	snp_rs2025072_0w_f	rs2025072	AIMS	92627138	0.554	0.715	0.16099	2	G2
14	snp_rs9671457_0w_r	rs9671457	AIMS	95016836	0.170	0.239	0.06835	1	G2
14	snp_rs9323913_0w_f	rs9323913	Both	95147310	0.511	0.927	0.41648	5	G2
14	snp_rs11624336_0w_f	rs11624336	AncAm	97193512	0.461	0.841	0.37957	4	G2
14	snp_rs1459134_1wa0_f	rs1459134	AncAm	98814319	0.172	0.175	0.00316	1	G2
14	snp_rs1024350_0w_r	rs1024350	AIMS	107141122	0.337	0.560	0.22308	3	G2
15	snp_rs999842_0w_f	rs999842	AIMS	23000272	0.467	0.396	0.07078	1	G1
15	snp_rs10519445_0w_f	rs10519445	AIMS	24106808	0.428	0.607	0.17925	2	G2
15	snp_rs2739818_0w_f	rs2739818	AIMS	25551253	0.144	0.029	0.11492	2	G1
15	snp_rs1432133_0w_f	rs1432133	AncAm	27228346	0.457	0.962	0.50426	6	G2
15	snp_rs1947745_0w_r	rs1947745	AIMS	27866498	0.112	0.053	0.05818	1	G1
15	snp_rs1800404_0w_r	rs1800404	AIMS	28235773	0.476	0.569	0.09365	1	G2
15	snp_rs1828774_0w_r	rs1828774	AncAm	29013164	0.512	0.960	0.44867	5	G2
15	snp_rs11634609_0w_r	rs11634609	AIMS	30213412	0.299	0.536	0.23656	3	G2
15	snp_rs2611605_0w_f	rs2611605	AIMS	32441633	0.854	0.942	0.08786	1	G2
15	snp_rs12440440_0w_r	rs12440440	AncAm	34041896	0.534	0.867	0.33329	4	G2
15	snp_rs11854468_0w_f	rs11854468	AIMS	35450549	0.944	1.000	0.05605	1	G2
15	snp_rs9920881_0w_f	rs9920881	AncAm	35961254	0.389	0.916	0.52621	6	G2
15	snp_rs16962252_0w_f	rs16962252	AncAm	36290481	0.309	0.621	0.31144	4	G2
15	snp_rs12102256_0w_r	rs12102256	AncAm	37290293	0.167	0.883	0.71532	8	G2
15	snp_rs6496012_0w_35AG_f	rs6496012	AIMS	39150924	0.754	0.854	0.10044	2	G2
15	snp_rs8033957_0w_f	rs8033957	AncAm	40038433	0.255	0.756	0.50052	6	G2
15	snp_rs1153860_0w_f	rs1153860	AIMS	45636584	0.446	0.062	0.38458	4	G1
15	snp_rs4775379_0w_r	rs4775379	AIMS	46682794	0.136	0.516	0.38006	4	G2
15	snp_rs2009404_0w_r	rs2009404	AIMS	49216723	0.261	0.048	0.21296	3	G1
15	snp_rs2078139_0w_r	rs2078139	AIMS	50659834	0.718	0.610	0.10755	2	G1
15	snp_rs6493546_0w_r	rs6493546	AncAm	52506655	0.519	0.976	0.45729	5	G2
15	snp_rs1906433_0w_35AG_r	rs1906433	AIMS	53878948	0.375	0.166	0.20976	3	G1
15	snp_rs728244_0w_f	rs728244	AIMS	55816985	0.251	0.000	0.25121	3	G1
15	snp_rs8025686_0w_35CT_r	rs8025686	AIMS	56291175	0.305	0.206	0.09901	1	G1
15	snp_rs4646568_0w_f	rs4646568	AIMS	58344290	0.286	0.075	0.21128	3	G1
15	snp_rs16939937_0w_r	rs16939937	AncAm	58519664	0.305	0.852	0.54658	6	G2
15	snp_rs10519005_1wa0_r	rs10519005	AIMS	59711691	0.287	0.138	0.14959	2	G1
15	snp_rs16942820_0w_35AC_f	rs16942820	AIMS	60907766	0.014	0.000	0.01400	1	G1
15	snp_rs4270116_0w_f	rs4270116	AncAm	61704441	0.759	0.075	0.68405	7	G1
15	snp_rs881644_0w_f	rs881644	AIMS	63663411	0.142	0.000	0.14218	2	G1
15	hmrs11071746_f	rs11071746	AncAm	63733263	0.677	0.009	0.66870	7	G1
15	snp_rs11633399_0w_r	rs11633399	AncAm	64962630	0.428	0.000	0.42806	5	G1
15	snp_rs4255740_0w_f	rs4255740	AIMS	66772007	0.417	0.339	0.07716	1	G1
15	snp_rs4489954_0w_r	rs4489954	AncAm	68072075	0.433	0.006	0.42686	5	G1
15	snp_rs2292745_0w_r	rs2292745	AIMS	68624290	0.800	0.329	0.47134	5	G1
15	snp_rs6495018_0w_f	rs6495018	AIMS	73097018	0.000	0.233	0.23255	3	G2
15	snp_rs3784556_0w_f	rs3784556	AIMS	74332188	0.056	0.000	0.05577	1	G1
15	snp_rs4886944_0w_f	rs4886944	AIMS	78143499	0.209	0.134	0.07479	1	G1
15	snp_rs938682_0w_f	rs938682	AncAm	78896547	0.408	0.983	0.57433	6	G2
15	snp_rs3816282_0w_f	rs3816282	AIMS	79350840	0.070	0.000	0.06988	1	G1
15	snp_rs10519292_0w_f	rs10519292	AIMS	81077470	0.086	0.383	0.29726	3	G2
15	snp_rs1501371_0w_r	rs1501371	AIMS	82460440	0.417	0.887	0.47000	5	G2
15	snp_rs698486_0w_f	rs698486	AIMS	84058592	0.231	0.311	0.08057	1	G2
15	snp_rs7178655_0w_r	rs7178655	AncAm	84764241	0.320	0.925	0.60568	7	G2
15	snp_rs10520589_0w_r	rs10520589	AncAm	86010345	0.000	0.642	0.64208	7	G2
15	snp_rs2448928_0w_r	rs2448928	AIMS	87119340	0.123	0.286	0.16301	2	G2
15	snp_rs387727_0w_r	rs387727	AIMS	89640855	0.352	0.072	0.28002	3	G1
15	snp_rs8035124_0w_r	rs8035124	AIMS	92105708	0.646	0.201	0.44486	5	G1
15	snp_rs17733968_0w_f	rs17733968	AncAm	95678494	0.256	0.608	0.35153	4	G2
15	snp_rs8040452_0w_r	rs8040452	AIMS	96197257	0.483	0.198	0.28440	3	G1
15	snp_rs1510058_0w_r	rs1510058	AIMS	99855009	0.079	0.255	0.17539	2	G2
15	snp_rs11247229_0w_r	rs11247229	AncAm	101195178	0.227	0.878	0.65076	7	G2
15	snp_rs8043261_0w_f	rs8043261	AIMS	101279066	0.317	0.941	0.62421	7	G2
16	hmrs2072986_f	rs2072986	AIMS	1691106	0.233	0.640	0.40644	5	G2
16	snp_rs1891325_0w_f	rs1891325	AIMS	6432408	0.152	0.000	0.15215	2	G1
16	snp_rs4559892_0w_r	rs4559892	AncAm	7987743	0.623	0.275	0.34763	4	G1
16	snp_rs9937557_0w_f	rs9937557	AIMS	8304390	0.272	0.762	0.48940	5	G2
16	snp_rs9927623_0w_f	rs9927623	AncAm	8304438	0.277	0.766	0.48922	5	G2
16	snp_rs896401_0w_f	rs896401	AIMS	8454655	0.760	0.098	0.66229	7	G1
16	snp_rs12929500_0w_f	rs12929500	AncAm	10692832	0.450	0.731	0.28105	3	G2
16	snp_rs4781011_0w_f	rs4781011	AIMS	10975311	0.498	0.168	0.32973	4	G1
16	snp_rs7185307_0w_r	rs7185307	AncAm	12046899	0.374	0.911	0.53662	6	G2
16	snp_rs276639_0w_r	rs276639	AIMS	13179016	0.776	0.495	0.28032	3	G1
16	snp_rs30237_0w_r	rs30237	AIMS	14374645	0.238	0.142	0.09646	1	G1
16	snp_rs11647711_0w_f	rs11647711	AIMS	17568525	0.000	0.027	0.02667	1	G2
16	snp_rs7197580_0w_r	rs7197580	AncAm	19278625	0.494	0.091	0.40263	5	G1
16	snp_rs13333521_0w_f	rs13333521	AIMS	19904082	0.089	0.121	0.03281	1	G2
16	snp_rs9930730_0w_r	rs9930730	AIMS	21017270	0.113	0.382	0.26885	3	G2
16	hmrs2238517_r	rs2238517	AIMS	24339933	0.174	0.379	0.20578	3	G2
16	snp_rs7205880_0w_f	rs7205880	AIMS	25851191	0.189	0.556	0.36646	4	G2
16	snp_rs4073229_0w_r	rs4073229	AncAm	25995418	0.463	0.104	0.35939	4	G1
16	snp_rs7350820_0w_r	rs7350820	AncAm	26474984	0.479	0.000	0.47907	5	G1
16	snp_rs8046736_0w_r	rs8046736	AIMS	26943239	0.222	0.126	0.09569	1	G1
16	snp_rs9934579_0w_f	rs9934579	AIMS	30474856	0.098	0.000	0.09773	1	G1
16	snp_rs12446160_0w_f	rs12446160	AIMS	48358368	0.588	0.595	0.00794	1	G2
16	snp_rs4785358_0w_f	rs4785358	AIMS	49839782	0.807	0.803	0.00422	1	G1
16	hmrs4347621_r	rs4347621	AIMS	51683901	0.210	0.000	0.21039	3	G1
16	snp_rs3112586_1wa0_r	rs3112586	AIMS	52684044	0.045	0.000	0.04486	1	G1
16	snp_rs1420228_0w_r	rs1420228	AIMS	55485173	0.068	0.010	0.05810	1	G1
16	snp_rs9937047_0w_f	rs9937047	AIMS	58145585	0.599	0.302	0.29728	3	G1
16	snp_rs4238802_0w_f	rs4238802	AIMS	59147942	0.362	0.330	0.03237	1	G1
16	snp_rs9927943_0w_r	rs9927943	AIMS	60574394	0.387	0.713	0.32595	4	G2
16	snp_rs1834072_0w_r	rs1834072	AIMS	62398175	0.609	0.412	0.19710	2	G1
16	snp_rs8060376_0w_r	rs8060376	AIMS	63798930	0.726	0.912	0.18638	2	G2
16	snp_rs818386_0w_f	rs818386	AIMS	65406708	0.737	0.289	0.44796	5	G1
16	snp_rs8063013_0w_f	rs8063013	AIMS	72356899	0.222	0.081	0.14060	2	G1
16	snp_rs825842_0w_f	rs825842	AncAm	73525237	0.615	0.122	0.49314	5	G1
16	snp_rs8054840_0w_r	rs8054840	AIMS	74850912	0.682	0.642	0.03946	1	G1
16	hmrs2288041_f	rs2288041	AncAm	74921446	0.682	0.131	0.55162	6	G1
16	snp_rs7501326_1wa1_f	rs7501326	AIMS	75942896	0.124	0.402	0.27846	3	G2
16	snp_rs17770042_0w_f	rs17770042	AIMS	77345811	0.008	0.669	0.66045	7	G2
16	snp_rs3751834_0w_r	rs3751834	AIMS	79016408	0.089	0.308	0.21888	3	G2
16	snp_rs8043824_0w_f	rs8043824	AIMS	79847142	0.272	0.290	0.01810	1	G2
16	snp_rs12917698_0w_f	rs12917698	AncAm	80335572	0.370	1.000	0.62973	7	G2
16	snp_rs1110789_0w_f	rs1110789	AIMS	80440301	0.276	0.225	0.05091	1	G1
16	snp_rs4889327_0w_r	rs4889327	AIMS	81536652	0.557	0.811	0.25330	3	G2
16	snp_rs25452_0w_f	rs25452	AIMS	82618604	0.749	0.375	0.37337	4	G1
16	snp_rs8063330_0w_f	rs8063330	AncAm	82627952	0.743	0.366	0.37652	4	G1
16	snp_rs11150519_0w_f	rs11150519	AncAm	82934307	0.481	0.654	0.17254	2	G2
16	snp_rs4843467_0w_r	rs4843467	AncAm	86883820	0.298	0.852	0.55397	6	G2
16	snp_rs6539986_0w_r	rs6539986	AIMS	86969210	0.242	0.362	0.11992	2	G2
17	snp_rs7215135_0w_f	rs7215135	AIMS	9164548	0.167	0.170	0.00322	1	G2
17	snp_rs758112_0w_r	rs758112	AIMS	10898453	0.502	0.817	0.31436	4	G2
17	snp_rs8067785_0w_r	rs8067785	AIMS	11930981	0.163	0.047	0.11591	2	G1
17	hmrs765101_r	rs765101	AIMS	13653880	0.272	0.037	0.23499	3	G1
17	snp_rs3930821_0w_f	rs3930821	AncAm	20697797	0.800	0.221	0.57866	6	G1
17	snp_rs7220080_0w_r	rs7220080	AIMS	25589184	0.462	0.694	0.23203	3	G2
17	snp_rs12601963_0w_f	rs12601963	AIMS	28246358	0.364	0.326	0.03821	1	G1
17	snp_rs3091326_0w_r	rs3091326	AncAm	32601183	0.299	0.892	0.59316	6	G2
17	snp_rs2411189_0w_f	rs2411189	AIMS	35009883	0.219	0.314	0.09517	1	G2
17	snp_rs6607302_0w_r	rs6607302	AncAm	36194230	0.662	0.107	0.55549	6	G1
17	snp_rs16965350_0w_r	rs16965350	AncAm	36614563	0.678	0.098	0.57915	6	G1
17	snp_rs1859251_0w_f	rs1859251	AIMS	39096618	1.000	0.429	0.57128	6	G1
17	hmrs8081000_f	rs8081000	AIMS	41715233	0.428	0.306	0.12140	2	G1
17	snp_rs11870879_0w_r	rs11870879	AIMS	47747648	0.652	0.723	0.07070	1	G2
17	snp_rs9303599_0w_r	rs9303599	AIMS	50577944	0.416	0.045	0.37112	4	G1
17	snp_rs7215254_0w_f	rs7215254	AIMS	51885944	0.285	0.022	0.26314	3	G1
17	snp_rs6504989_0w_r	rs6504989	AIMS	53734024	0.182	0.258	0.07655	1	G2
17	snp_rs7211655_0w_35CT_r	rs7211655	Both	55855595	0.422	0.917	0.49532	5	G2
17	snp_rs7210832_0w_f	rs7210832	AIMS	57608214	0.379	0.248	0.13125	2	G1
17	hmrs758467_f	rs758467	AIMS	59314257	0.248	0.163	0.08482	1	G1
17	snp_rs196948_0w_r	rs196948	AIMS	62157307	0.683	0.480	0.20269	3	G1
17	snp_rs2869836_0w_r	rs2869836	AncAm	63333704	0.776	0.200	0.57539	6	G1
17	snp_rs2072268_0w_f	rs2072268	AIMS	66303352	0.357	0.120	0.23638	3	G1
17	snp_rs8071270_0w_r	rs8071270	AIMS	69395948	0.192	0.181	0.01097	1	G1
17	snp_rs7217976_0w_r	rs7217976	AncAm	69481277	0.636	0.308	0.32873	4	G1
17	snp_rs6501805_0w_f	rs6501805	AncAm	73455928	0.264	0.690	0.42601	5	G2
17	snp_rs9892081_0w_r	rs9892081	AncAm	78484484	0.451	0.750	0.29823	3	G2
18	snp_rs2606246_0w_r	rs2606246	AIMS	678847	0.361	0.411	0.05009	1	G2
18	snp_rs11081150_0w_r	rs11081150	AIMS	4786165	0.101	0.000	0.10104	2	G1
18	snp_rs17519184_0w_f	rs17519184	Both	6986714	0.509	0.176	0.33331	4	G1
18	snp_rs400839_0w_f	rs400839	AIMS	8660149	0.721	0.466	0.25545	3	G1
18	snp_rs206501_0w_r	rs206501	AIMS	10426035	0.446	0.113	0.33366	4	G1
18	snp_rs4797585_0w_f	rs4797585	AncAm	11911143	0.500	0.271	0.22986	3	G1
18	snp_rs7235524_0w_f	rs7235524	AIMS	19718263	0.577	0.398	0.17923	2	G1
18	snp_rs12970899_0w_f	rs12970899	AIMS	21148863	0.103	0.050	0.05319	1	G1
18	snp_rs1786153_0w_r	rs1786153	AIMS	22498170	0.528	0.633	0.10550	2	G2
18	snp_rs11564361_0w_r	rs11564361	AIMS	25904868	0.266	0.106	0.16017	2	G1
18	snp_rs1464612_0w_r	rs1464612	AIMS	25915140	0.457	0.651	0.19463	2	G2
18	snp_rs8085075_0w_f	rs8085075	AIMS	33340249	0.314	0.352	0.03835	1	G2
18	snp_rs507163_0w_f	rs507163	AncAm	33907628	0.444	0.922	0.47752	5	G2
18	snp_rs978807_0w_r	rs978807	AIMS	36898459	0.154	0.124	0.02953	1	G1
18	snp_rs10502736_0w_r	rs10502736	AncAm	37970495	0.327	0.749	0.42207	5	G2
18	snp_rs2082325_0w_r	rs2082325	AIMS	39418682	0.478	0.215	0.26293	3	G1
18	snp_rs669898_0w_f	rs669898	Both	40500066	0.578	0.970	0.39167	4	G2
18	snp_rs4890673_0w_f	rs4890673	AIMS	44134616	0.056	0.199	0.14261	2	G2
18	snp_rs1393515_0w_r	rs1393515	AIMS	45206799	0.420	0.479	0.05897	1	G2
18	snp_rs506426_0w_r	rs506426	AIMS	46849333	0.112	0.000	0.11180	2	G1
18	snp_rs1954882_0w_r	rs1954882	AIMS	48290668	0.197	0.100	0.09690	1	G1
18	snp_rs7506909_0w_r	rs7506909	AncAm	50620087	0.311	0.133	0.17792	2	G1
18	snp_rs1451949_0w_r	rs1451949	AIMS	52290083	0.540	0.555	0.01534	1	G2
18	snp_rs6567038_0w_r	rs6567038	AncAm	56637843	0.178	0.581	0.40256	5	G2
18	snp_rs881728_0w_r	rs881728	AIMS	59333108	0.767	0.509	0.25809	3	G1
18	snp_rs8083511_0w_f	rs8083511	AIMS	60028655	0.277	0.348	0.07157	1	G2
18	snp_rs4941246_0w_f	rs4941246	AIMS	61802304	0.222	0.170	0.05133	1	G1
18	snp_rs17079195_0w_f	rs17079195	AIMS	66137894	0.096	0.170	0.07457	1	G2
18	snp_rs4891825_0w_r	rs4891825	AIMS	67867663	0.083	0.009	0.07358	1	G1
18	snp_rs2685428_0w_r	rs2685428	AncAm	69487009	0.635	0.980	0.34523	4	G2
18	snp_rs4479346_0w_f	rs4479346	AIMS	69550009	0.276	0.260	0.01558	1	G1
18	snp_rs11151863_0w_f	rs11151863	AIMS	71119879	0.667	0.794	0.12727	2	G2
18	snp_rs17211623_0w_r	rs17211623	AIMS	73203543	0.853	0.631	0.22245	3	G1
18	snp_rs12970236_0w_f	rs12970236	AIMS	78007784	0.578	0.850	0.27172	3	G2
19	snp_rs2434444_1wa0_f	rs2434444	AIMS	7466037	0.712	0.597	0.11518	2	G1
19	snp_rs8111998_0w_r	rs8111998	AIMS	22741675	0.204	0.085	0.11901	2	G1
19	snp_rs10420543_0w_f	rs10420543	AncAm	35897918	0.468	0.838	0.36957	4	G2
19	snp_rs7976_0w_f	rs7976	AIMS	35978299	0.888	1.000	0.11155	2	G2
19	snp_rs8109818_0w_f	rs8109818	AIMS	41460621	0.158	0.504	0.34643	4	G2
19	snp_rs1988065_0w_f	rs1988065	AIMS	43374601	0.132	0.334	0.20269	3	G2
19	snp_rs2061332_0w_r	rs2061332	AncAm	44613661	0.241	0.778	0.53689	6	G2
19	snp_rs4802234_0w_f	rs4802234	AIMS	45222600	0.439	0.379	0.06025	1	G1
19	hmrs10409570_r	rs10409570	AIMS	50945411	0.056	0.000	0.05622	1	G1
19	snp_rs10500308_1wa0_r	rs10500308	AIMS	52108930	0.332	0.696	0.36322	4	G2
19	snp_rs7507442_0w_f	rs7507442	AncAm	53278953	0.384	0.046	0.33748	4	G1
20	snp_rs6515824_0w_r	rs6515824	AIMS	97122	0.161	0.012	0.14886	2	G1
20	snp_rs805764_0w_f	rs805764	AIMS	5676783	0.204	0.261	0.05727	1	G2
20	hmrs6104567_r	rs6104567	AIMS	10195433	0.441	0.236	0.20460	3	G1
20	snp_rs1041200_0w_f	rs1041200	AIMS	10979521	0.234	0.198	0.03594	1	G1
20	snp_rs4813048_0w_r	rs4813048	AncAm	11169603	0.666	0.918	0.25130	3	G2
20	snp_rs1321936_0w_r	rs1321936	AncAm	12826999	0.338	0.028	0.30955	4	G1
20	snp_rs8116153_0w_r	rs8116153	AIMS	13234124	0.028	0.000	0.02797	1	G1
20	snp_rs1884783_0w_f	rs1884783	AIMS	18725724	0.484	0.252	0.23216	3	G1
20	snp_rs6081756_0w_r	rs6081756	AIMS	19804868	0.582	0.715	0.13291	2	G2
20	snp_rs6137241_0w_f	rs6137241	AIMS	21049362	0.212	0.185	0.02686	1	G1
20	snp_rs6050867_0w_r	rs6050867	AIMS	25730968	0.452	0.344	0.10832	2	G1
20	snp_rs2057262_0w_r	rs2057262	AIMS	31687132	0.493	0.611	0.11871	2	G2
20	snp_rs6088466_0w_r	rs6088466	AIMS	32913534	0.309	1.000	0.69134	7	G2
20	snp_rs224371_0w_f	rs224371	AIMS	34074831	0.445	0.117	0.32870	4	G1
20	snp_rs6126462_0w_f	rs6126462	AIMS	36525376	0.496	0.882	0.38637	4	G2
20	snp_rs6129532_0w_f	rs6129532	AIMS	38884188	0.774	0.868	0.09447	1	G2
20	snp_rs4812831_0w_r	rs4812831	Both	43018260	0.325	0.788	0.46332	5	G2
20	snp_rs6032343_0w_r	rs6032343	AIMS	44204290	0.272	0.272	0.00028	1	G1
20	snp_rs477627_0w_r	rs477627	AIMS	48180058	0.111	0.000	0.11124	2	G1
20	snp_rs2041317_0w_r	rs2041317	AIMS	52363607	0.144	0.157	0.01331	1	G2
20	snp_rs3907047_0w_f	rs3907047	AIMS	54000914	0.367	0.585	0.21801	3	G2
20	snp_rs2426689_0w_f	rs2426689	AncAm	55369594	0.347	0.000	0.34669	4	G1
20	snp_rs1877751_0w_r	rs1877751	Both	57967906	0.521	0.016	0.50502	6	G1
20	snp_rs487656_0w_r	rs487656	AncAm	58790814	0.560	0.302	0.25864	3	G1
20	snp_rs6071491_0w_r	rs6071491	AIMS	59684581	0.125	0.000	0.12524	2	G1
21	snp_rs2205254_0w_r	rs2205254	AIMS	15725642	0.490	0.391	0.09849	1	G1
21	snp_rs8127304_0w_r	rs8127304	AIMS	16956866	0.255	0.041	0.21367	3	G1
21	snp_rs2776068_0w_f	rs2776068	AIMS	18877791	0.367	0.802	0.43467	5	G2
21	snp_rs1735899_0w_r	rs1735899	AIMS	21812615	0.408	0.230	0.17749	2	G1
21	snp_rs2826910_0w_f	rs2826910	AIMS	22983216	0.136	0.003	0.13293	2	G1
21	snp_rs2828417_0w_f	rs2828417	AIMS	25050091	0.013	0.704	0.69126	7	G2
21	snp_rs2830051_0w_f	rs2830051	AncAm	27465355	0.470	0.766	0.29596	3	G2
21	snp_rs2832643_0w_f	rs2832643	AIMS	31528217	0.283	0.131	0.15209	2	G1
21	snp_rs2833256_0w_r	rs2833256	AncAm	32444485	0.808	0.247	0.56113	6	G1
21	hmrs558059_f	rs558059	AIMS	32810899	0.754	0.119	0.63481	7	G1
21	hmrs2300395_r	rs2300395	AIMS	36245198	0.166	0.037	0.12891	2	G1
21	snp_rs1573303_0w_r	rs1573303	AIMS	37312170	0.229	0.334	0.10496	2	G2
21	snp_rs2836181_1wa0_r	rs2836181	AIMS	39555810	0.074	0.078	0.00397	1	G2
21	snp_rs2836824_0w_f	rs2836824	AIMS	40380823	0.255	0.211	0.04340	1	G1
21	snp_rs2187239_0w_r	rs2187239	Both	43420555	0.480	0.838	0.35828	4	G2
21	snp_rs762421_0w_r	rs762421	Both	45615561	0.642	0.936	0.29356	3	G2
21	snp_rs2839367_0w_f	rs2839367	AIMS	48050388	0.206	0.252	0.04595	1	G2
22	hmrs5748919_f	rs5748919	AncAm	17644131	0.536	0.084	0.45226	5	G1
22	snp_rs658793_0w_r	rs658793	AIMS	20996595	0.804	0.413	0.39047	4	G1
22	snp_rs5754727_1wa1_f	rs5754727	AIMS	22057273	0.641	0.393	0.24747	3	G1
22	snp_rs5762632_0w_f	rs5762632	AIMS	28847006	0.196	0.325	0.12928	2	G2
22	snp_rs134678_0w_r	rs134678	AncAm	29503955	0.548	0.956	0.40789	5	G2
22	snp_rs136245_0w_r	rs136245	AIMS	31208544	0.528	0.962	0.43355	5	G2
22	hmrs6518754_f	rs6518754	AncAm	32097775	0.502	1.000	0.49830	5	G2
22	snp_rs5754506_0w_f	rs5754506	AIMS	33683735	0.194	0.000	0.19401	2	G1
22	snp_rs909704_0w_r	rs909704	AIMS	35899384	0.708	0.236	0.47168	5	G1
22	snp_rs5757362_1wa1_f	rs5757362	Both	39306080	0.568	0.000	0.56839	6	G1
22	snp_rs470113_0w_r	rs470113	Both	40729614	0.340	0.762	0.42101	5	G2

Chr, chromosome; SNP-Array ID, single nucleotide polymorphism identifier in the array; dbSNP, single nucleotide polymorphism identifier; Pos, position; Six groups were formed for cross validation analysis, which are shown in the Frequency group column; In the admixture analysis, the minimum cross-validation error using the ancestry markers for the population of this study was for K = 2, which is represented by the partition of the frequency allele of each SNP in the two groups (G1 and G2); AF G1 is the allelic frequency for group 1; AF G2 is the allelic frequency for group 2.

**Table 2:** Description of candidate SNP nominally associated with T2 susceptible to be replicated in future studies.

Chr	SNP-Array ID	GenDist	PhysPosit	allele	T2D-G	NORMAL	OR	LowerCL	UpperCL	ProbGenotype	ProbAllele	ProbTrend	lpa	lpg	lpt
1	snp_rs17128450_0w_f	0.9262	66613479	G/T	47	44	4.958	2.368	10.380	0.0045356	0.0000086	0.0015298	5.07	2.34	2.82
1	snp_rs34055463_0w_f	0.9778	70948709	C/T	47	45	0.063	0.014	0.278	0.0000350	0.0000035	0.0000119	5.45	4.46	4.93
1	hmrs11163314_f	1.0676	81957297	A/G	43	44	0.077	0.023	0.267	0.0009297	0.0000010	0.0004885	5.98	3.03	3.31
1	snp_rs2104211_0w_r	1.7913	177651228	A/C	47	45	4.229	2.220	8.056	0.0001963	0.0000067	0.0000415	5.17	3.71	4.38
2	snp_rs4669186_0w_f	0.1779	7485817	C/T	46	45	8.272	3.031	22.575	0.0001207	0.0000036	0.0000657	5.45	3.92	4.18
2	snp_rs10754950_0w_f	0.1788	7512594	A/G	47	45	0.124	0.049	0.314	0.0000552	0.0000010	0.0000237	5.99	4.26	4.62
2	snp_rs13406490_0w_f	0.2288	8968446	A/G	47	45	0.256	0.139	0.475	0.0000189	0.0000099	0.0000032	5.00	4.72	5.49
2	snp_rs7590835_1wa1_r	0.6377	38893950	C/T	47	45	8.092	2.163	30.268	0.0005497	0.0000010	0.0005497	5.99	3.26	3.26
2	snp_rs1861427_1wa0_f	0.6489	40387489	A/G	47	45	0.250	0.134	0.464	0.0002392	0.0000074	0.0000453	5.13	3.62	4.34
2	snp_rs7566955_0w_f	1.5218	140817541	C/T	47	45	0.190	0.095	0.378	0.0000599	0.0000008	0.0000113	6.07	4.22	4.95
2	snp_rs6436247_0w_r	2.2382	222150408	A/G	47	45	0.081	0.024	0.280	0.0000047	0.0000017	0.0000013	5.76	5.33	5.88
2	snp_rs17351792_0w_f	2.2382	222152611	C/T	47	45	0.109	0.036	0.329	0.0000368	0.0000061	0.0000096	5.21	4.43	5.02
3	snp_rs2347654_0w_f	0.3751	16884808	C/T	47	44	4.165	2.220	7.813	0.0001057	0.0000054	0.0000375	5.27	3.98	4.43
3	KG_3_55243502_f	0.7568	55268462	C/T	47	45	4.838	2.535	9.235	0.0000378	0.0000008	0.0000066	6.08	4.42	5.18
3	snp_rs6804524_0w_f	0.8737	63770138	A/G	47	45	26.571	3.482	202.757	0.0001911	0.0000064	0.0000764	5.19	3.72	4.12
3	snp_rs7609759_0w_r	0.8738	63771504	A/G	47	45	14.882	3.384	65.448	0.0001613	0.0000070	0.0000521	5.15	3.79	4.28
3	snp_rs7429997_0w_f	0.9607	71235982	C/T	47	45	0.239	0.128	0.447	0.0002347	0.0000045	0.0000451	5.34	3.63	4.35
3	snp_rs6800542_0w_r	1.6690	162276129	C/T	46	43	0.229	0.121	0.433	0.0000612	0.0000035	0.0000175	5.46	4.21	4.76
3	snp_rs7646242_0w_r	1.6690	162281636	C/T	47	45	0.206	0.109	0.388	0.0000159	0.0000005	0.0000044	6.30	4.80	5.36
3	snp_rs2088315_0w_r	1.6693	162382517	A/G	47	45	0.190	0.095	0.378	0.0000219	0.0000008	0.0000048	6.07	4.66	5.32
3	snp_rs16861676_0w_r	1.9705	186903502	C/T	47	44	5.778	2.662	12.541	0.0000469	0.0000025	0.0000136	5.61	4.33	4.87
3	snp_rs3864095_0w_f	1.9719	186937037	A/G	47	45	5.720	2.755	11.874	0.0000084	0.0000008	0.0000055	6.09	5.08	5.26
3	snp_rs3774264_0w_r	1.9719	186937466	C/T	47	45	0.199	0.096	0.413	0.0000533	0.0000058	0.0000295	5.24	4.27	4.53
4	KG_4_16945820_f	0.3321	17336722	C/T	47	45	0.256	0.130	0.504	0.0000071	0.0000484	0.0000903	4.31	5.15	4.04
4	b36_4_16984049_f	0.3331	17374951	C/T	47	45	0.233	0.124	0.436	0.0000172	0.0000032	0.0000252	5.50	4.76	4.60
4	snp_rs1670754_0w_r	0.5211	32264997	A/G	47	45	7.697	3.207	18.470	0.0000299	0.0000005	0.0000056	6.29	4.52	5.25
4	KG_4_152224870_f	1.4520	152005420	C/T	47	44	5.185	2.443	11.005	0.0000968	0.0000064	0.0000171	5.19	4.01	4.77
4	snp_rs4602464_0w_f	1.6689	172775756	A/C	47	45	0.191	0.092	0.399	0.0001199	0.0000036	0.0000262	5.44	3.92	4.58
4	KG_4_187322882_f	1.9592	187085888	A/G	47	45	8.654	2.873	26.066	0.0000389	0.0000120	0.0000094	4.92	4.41	5.02
5	snp_rs17250927_0w_r	0.2064	8100597	A/G	47	45	0.104	0.035	0.311	0.0000160	0.0000031	0.0000043	5.51	4.80	5.36
5	KG_5_52315617_f	0.6821	52279860	C/T	46	45	0.121	0.044	0.330	0.0000001	0.0000036	0.0000001	5.45	6.89	6.89
5	snpind_rs10940293_0w_f	0.6843	52475201	A/C	47	45	4.555	2.419	8.579	0.0000032	0.0000014	0.0000030	5.84	5.50	5.52
5	snp_rs898219_0w_f	0.8296	71240138	G/T	47	45	4.005	2.155	7.443	0.0000879	0.0000074	0.0000155	5.13	4.06	4.81
5	snp_rs7448059_0w_r	1.8146	169918840	C/T	47	45	4.577	2.274	9.214	0.0000570	0.0000096	0.0000266	5.02	4.24	4.58
6	snp_rs850176_0w_r	0.2093	8573040	G/T	47	45	7.231	2.635	19.846	0.0000247	0.0000196	0.0000050	4.71	4.61	5.30
6	KG_6_30027077_r	0.5107	29919098	A/G/	45	38	0.145	0.059	0.356	0.0024064	0.0000048	0.0010993	5.32	2.62	2.96
7	snp_rs12056190_0w_r	0.0764	3307922	A/G	47	45	0.231	0.118	0.450	0.0000297	0.0000091	0.0000050	5.04	4.53	5.30
7	snp_rs2049477_0w_r	0.0764	3312870	A/C	47	45	0.175	0.084	0.364	0.0000130	0.0000009	0.0000021	6.04	4.89	5.67
7	snp_rs11767642_1wa1_r	0.2541	13274710	G/T	47	44	0.211	0.108	0.410	0.0000284	0.0000022	0.0000081	5.66	4.55	5.09
7	snp_rs33911118_0w_f	0.5447	34873776	A/G	47	45	4.115	2.116	8.001	0.0000031	0.0000176	0.0000120	4.76	5.51	4.92
7	snp_rs497459_0w_f	0.7163	49838522	C/T	45	43	6.683	2.750	16.244	0.0044216	0.0000052	0.0010485	5.28	2.35	2.98
7	snp_rs2525539_0w_f	1.0935	99545309	A/G	47	45	0.035	0.005	0.269	0.0000084	0.0000033	0.0000022	5.49	5.08	5.65
7	snp_rs759550_1wa0_f	1.1804	107932915	C/T	45	44	0.243	0.130	0.455	0.0001246	0.0000062	0.0000284	5.20	3.90	4.55
7	snp_rs10261386_0w_f	1.2441	120711855	C/T	47	45	0.149	0.068	0.323	0.0000079	0.0000003	0.0000055	6.59	5.10	5.26
7	snp_rs10266232_0w_r	1.3478	131381498	A/C	47	45	0.218	0.109	0.440	0.0000252	0.0000096	0.0000170	5.02	4.60	4.77
7	snp_rs367567_0w_f	1.6314	151064422	A/G	47	45	0.240	0.127	0.450	0.0001195	0.0000055	0.0000222	5.26	3.92	4.65
7	b36_7_150700229_r	1.6317	151069296	A/G	47	45	4.091	2.172	7.705	0.0000382	0.0000079	0.0000120	5.10	4.42	4.92
8	KG_8_6674089_r	0.1604	6686679	C/T	47	45	0.277	0.144	0.531	0.0000053	0.0000767	0.0000639	4.11	5.27	4.19
8	snp_rs4841579_0w_f	0.2196	11516592	G/T	47	45	0.181	0.085	0.386	0.0000417	0.0000028	0.0000114	5.55	4.38	4.94
8	b36_8_11660645_r	0.2209	11623236	A/G	47	44	0.227	0.122	0.423	0.0000462	0.0000018	0.0000080	5.74	4.33	5.10
8	snp_rs13271261_0w_r	0.2563	13898663	C/T	47	45	0.221	0.116	0.420	0.0000833	0.0000022	0.0000161	5.66	4.08	4.79
9	snp_rs7865839_0w_r	0.0767	3648561	C/T	43	40	0.000	.	.	0.0011453	0.0000042	0.0011453	5.37	2.94	2.94
9	snp_rs10814931_0w_r	0.0957	4319915	C/T	47	45	0.176	0.081	0.383	0.0000178	0.0000032	0.0000075	5.50	4.75	5.13
9	KG_9_22147831_r	0.4349	22157831	A/G	47	45	0.095	0.032	0.281	0.0000217	0.0000008	0.0000061	6.08	4.66	5.21
9	snp_rs2812378_0w_35CT_r	0.5697	34710260	A/G	47	45	0.223	0.119	0.418	0.0000111	0.0000016	0.0000022	5.80	4.95	5.66
9	snp_rs327953_0w_f	1.0792	107965241	C/T	45	44	0.179	0.080	0.402	0.0000168	0.0000093	0.0000035	5.03	4.78	5.45
9	KG_9_107019007_f	1.0795	107979186	C/T	47	45	0.132	0.048	0.359	0.0000170	0.0000099	0.0000040	5.00	4.77	5.40
9	b36_9_136555163_f	1.5198	137415342	C/T	46	43	14.538	3.293	64.191	0.0053289	0.0000098	0.0015450	5.01	2.27	2.81
10	b36_10_24533332_r	0.4772	24493326	C/T	47	45	3.933	2.127	7.275	0.0001576	0.0000085	0.0000717	5.07	3.80	4.14
10	snp_rs1219563_0w_f	0.6240	36526084	C/T	47	45	0.259	0.140	0.477	0.0000811	0.0000099	0.0000151	5.00	4.09	4.82
10	snp_rs12781176_0w_f	0.6240	36528990	C/T	47	42	0.248	0.132	0.466	0.0001675	0.0000096	0.0000340	5.02	3.78	4.47
10	snp_rs1219625_0w_f	0.6241	36562418	A/G	47	45	0.222	0.119	0.414	0.0000967	0.0000012	0.0000239	5.93	4.01	4.62
10	b36_10_36678431_r	0.6243	36638425	A/G	45	45	5.000	2.576	9.703	0.0000229	0.0000009	0.0000069	6.06	4.64	5.16
10	b36_10_71958308_r	0.8891	72288302	A/C	47	45	4.630	2.462	8.708	0.0000053	0.0000010	0.0000010	5.98	5.27	6.01
10	b36_10_71988061_f	0.8898	72318055	A/G	47	45	0.220	0.107	0.451	0.0000532	0.0000170	0.0000094	4.77	4.27	5.03
10	snp_rs1641689_0w_f	0.8911	72374748	A/G	47	45	5.345	2.449	11.669	0.0000155	0.0000083	0.0000030	5.08	4.81	5.52
10	snp_rs4980267_0w_r	1.4854	124936899	A/G	47	44	0.072	0.016	0.315	0.0000021	0.0000138	0.0000021	4.86	5.67	5.67
11	KG_11_37196045_f	0.5578	37239469	A/G	47	45	0.226	0.118	0.432	0.0000179	0.0000037	0.0000031	5.44	4.75	5.51
11	snp_rs11034036_0w_r	0.5578	37256190	C/T	47	45	0.211	0.109	0.407	0.0000044	0.0000016	0.0000009	5.79	5.35	6.03
11	snp_rs10836714_0w_f	0.5579	37275932	C/T	47	45	4.432	2.317	8.480	0.0000092	0.0000037	0.0000018	5.44	5.04	5.75
11	snp_rs10894376_0w_r	1.4483	131204890	A/C	47	45	4.154	2.194	7.867	0.0000697	0.0000073	0.0000157	5.13	4.16	4.81
14	snp_rs10498307_0w_r	0.2525	29945853	C/T	47	45	16.727	3.820	73.243	0.0001502	0.0000018	0.0000457	5.75	3.82	4.34
14	KG_14_29039733_r	0.2527	29969982	A/G	47	45	17.692	4.048	77.324	0.0000706	0.0000009	0.0000231	6.05	4.15	4.64
15	snp_rs2286946_0w_f	0.1274	27402547	A/G	47	45	0.214	0.109	0.423	0.0001034	0.0000040	0.0000186	5.39	3.99	4.73
15	snp_rs6497269_0w_f	0.1408	27706279	A/G	47	45	0.237	0.126	0.444	0.0001172	0.0000043	0.0000210	5.37	3.93	4.68
15	snp_rs904372_0w_r	0.1512	27929091	C/T	47	45	0.207	0.108	0.394	0.0000151	0.0000008	0.0000028	6.08	4.82	5.56
15	snp_rs11635657_0w_f	0.2264	31398082	C/T	46	45	6.077	2.965	12.454	0.0000042	0.0000002	0.0000007	6.68	5.38	6.19
15	KG_15_77358198_r	0.8268	79571143	A/G	47	45	6.252	3.002	13.019	0.0000010	0.0000002	0.0000002	6.67	6.00	6.74
15	snp_rs8033304_0w_r	0.8276	79610215	C/T	47	45	0.229	0.107	0.490	0.0000516	0.0000723	0.0000098	4.14	4.29	5.01
15	KG_15_96268527_f	1.2231	98467523	A/G	46	45	12.046	3.502	41.433	0.0000762	0.0000023	0.0000817	5.65	4.12	4.09
16	snp_rs43161_0w_f	0.2274	8382737	A/G	47	45	4.294	2.209	8.349	0.0004022	0.0000095	0.0000911	5.02	3.40	4.04
16	snp_rs29582_0w_r	0.2275	8385729	A/G	47	45	4.831	2.522	9.253	0.0001337	0.0000010	0.0000266	6.02	3.87	4.57
16	snp_rs4782995_0w_r	1.1620	84552655	A/G	47	45	4.108	2.195	7.688	0.0000508	0.0000061	0.0000177	5.21	4.29	4.75
16	b36_16_85055958_f	1.2260	86498457	A/G	47	45	0.187	0.086	0.408	0.0002529	0.0000083	0.0000494	5.08	3.60	4.31
17	b36_17_6055157_f	0.1660	6114433	C/T	47	44	7.063	2.565	19.451	0.0000391	0.0000272	0.0000080	4.56	4.41	5.10
17	b36_17_6880246_f	0.1966	6939522	C/T	47	45	3.991	2.138	7.452	0.0004077	0.0000090	0.0000882	5.05	3.39	4.05
17	b36_17_8535819_r	0.2211	8595094	C/T	47	45	7.167	3.099	16.572	0.0000095	0.0000006	0.0000019	6.25	5.02	5.72
17	snp_rs12939782_0w_f	0.2215	8604495	A/C	47	45	9.141	3.042	27.470	0.0001543	0.0000061	0.0000376	5.21	3.81	4.42
17	b36_17_33847507_r	0.6664	36593981	G/T	46	45	3.777	2.042	6.988	0.0000250	0.0000160	0.0000088	4.80	4.60	5.06
17	b36_17_33866474_r	0.6666	36612948	A/C	47	45	5.345	2.449	11.669	0.0002529	0.0000083	0.0000494	5.08	3.60	4.31
17	b36_17_41694512_f	0.7353	44338735	C/T	45	44	6.150	2.643	14.311	0.0026649	0.0000058	0.0006760	5.23	2.57	3.17
17	KG_17_78596289_r	1.3767	81003000	A/G	47	45	0.210	0.100	0.438	0.0000301	0.0000137	0.0000055	4.86	4.52	5.26
17	b36_17_78618682_r	1.3773	81025393	A/C	47	45	5.467	2.622	11.398	0.0000169	0.0000018	0.0000048	5.74	4.77	5.32
18	snp_rs663245_0w_f	0.4293	20046079	C/T	47	45	0.039	0.005	0.297	0.0046041	0.0000091	0.0012753	5.04	2.34	2.89
18	snp_rs3737395_0w_r	0.9778	67534710	A/G	47	45	0.104	0.035	0.311	0.0000113	0.0000031	0.0000032	5.52	4.95	5.50
19	b36_19_15863297_f	0.3855	16002297	C/T	47	44	0.102	0.034	0.303	0.0001162	0.0000022	0.0000279	5.66	3.93	4.55
19	KG_19_54080060_f	0.7949	49388248	C/T	44	44	4.788	2.438	9.402	0.0024739	0.0000026	0.0005531	5.59	2.61	3.26
20	snp_rs6068446_0w_r	0.8298	51676901	C/T	47	45	0.230	0.124	0.428	0.0000421	0.0000020	0.0000163	5.69	4.38	4.79
21	snp_rs4817543_0w_r	0.3754	34555503	C/T	47	45	0.190	0.097	0.374	0.0000133	0.0000006	0.0000030	6.26	4.88	5.52
21	snp_rs2838820_0w_f	0.7113	46646005	C/T	47	45	4.723	2.532	8.809	0.0000006	0.0000006	0.0000015	6.25	6.19	5.82
22	snp_rs134965_1wa1_r	0.2879	27552544	A/G	47	45	15.791	3.599	69.286	0.0003106	0.0000035	0.0000883	5.45	3.51	4.05
22	snp_rs136867_0w_r	0.3493	32272658	A/G	47	45	0.158	0.075	0.335	0.0000055	0.0000003	0.0000017	6.52	5.26	5.78
22	snp_rs8136613_0w_r	0.6867	49364828	C/T	47	45	5.000	2.518	9.930	0.0000102	0.0000017	0.0000024	5.76	4.99	5.62
24	snp_rs1914711_0w_r	1.1457	115288474	A/C	47	45	6.824	3.575	13.025	0.0000010	0.0000000	0.0000004	8.84	6.00	6.45
24	snp_rs5954118_0w_f	1.4660	139936715	C/A	47	45	4.524	2.379	8.604	0.0003521	0.0000022	0.0000761	5.66	3.45	4.12
24	snp_rs6636129_0w_r	1.4662	139944042	C/T	47	45	0.233	0.120	0.453	0.0008560	0.0000095	0.0002627	5.02	3.07	3.58
24	KG_X_140794831_f	1.5084	140967165	C/T	47	45	4.909	2.596	9.282	0.0000381	0.0000005	0.0000169	6.33	4.42	4.77

Chr, chromosome; SNP-Array ID, single nucleotide polymorphism identfiler in the array; GenDist, genetic distance; PhysPosit, physical position; T2D-G, type 2 diabetes group; OR, allele odds ratio for the first allele in allele column, if OR larger than 1 then the first allele is associated to diabetes; LowerCI, lower coefficient interval; Upper CI, upper coefficient interval; ProbGenotype, probability genotype model; ProbAllele, probability allele model; ProbTrend, probability trend model; lpa, logarithmic probability allele model; lpg, logarithmic probability genotype model; lpt, logarithmic probability trend model.

The analysis of 39 SNPs previously associated with T2D were replicated [[2], [3], [4], [5], [6], [7], [8]], and association with T2D (Table 3) was found for the rs4311394 in *ARL15* gene with a p = 0.001 using the genotype and Armitage trend tests [9]. In addition, the variants *HNF4A* rs1800961 and ADAMTS9-AS2 rs4607103 showed nominal association with this disease by the genotype and Armitage trend tests (p = 0.0340) and the allele test (p = 0.041).

**Table 3:** Summary Gene information of 39 SNP associated with T2D or metabolic traits.

Marker Summary										
Locus	Chr	Gene	SNP-Array ID	PP	N	PIC	Heterozygosity	Allelic diversity	HWE test	
									Chi-Square	PR > Chi-Square
M1	4	*WFS1*	snp_rs10010131_0w_r	6292915	94	0.308	0.383	0.3803	0.0048	0.9448
M2	3	*OGG1*	snp_rs1052133_1wa0_r	9798773	94	0.3567	0.5851	0.4646	6.3196	0.0119
M3	9	*CDKN2A/2B*	snp_rs10811661_0w_r	22134094	94	0.2157	0.2447	0.246	0.0026	0.9591
M4	6	*CDKAL1*	snp_rs10946398_0w_r	20661034	94	0.3482	0.4255	0.4491	0.2583	0.6113
M5	10	*HHEX*	snp_rs1111875_0w_r	94462882	94	0.3582	0.4894	0.4674	0.2074	0.6488
M6	8	*SLC30A8*	snp_rs11558471_0w_f	118185733	94	0.3172	0.3511	0.3954	1.1805	0.2773
M7	15	*ZFAND6*	snp_rs11634397_0w_r	80432222	94	0.375	0.383	0.5	5.1489	0.0233
M8	10	*TCF7L2*	snp_rs12255372_0w_f	114808902	94	0.1788	0.2021	0.1984	0.0323	0.8574
M9	8	*SLC30A8*	snp_rs13266634_1wa1_r	118184783	94	0.3201	0.3617	0.4002	0.8691	0.3512
M10	9	*CHCHD6*	snp_rs13292136_0w_r	81952128	91	0.2577	0.2418	0.3038	3.7979	0.0513
M11	16	*FT0*	snp_rs1421085_0w_r	53800954	94	0.1853	0.1915	0.2067	0.5062	0.4768
M12	7	*JAZF1*	snp_rs1635852_0w_r	28189411	93	0.3371	0.4731	0.4292	0.9746	0.3235
M13	16	*FTO*	snp_rs17817449_0w_r	53813367	94	0.1853	0.1915	0.2067	0.5062	0.4768
M14	20	*HNF4A*	snp_rs1800961_0w_f	43042364	94	0.1917	0.1809	0.2147	2.3418	0.1259
M15	3	*PPARG*	snp_rs1801282_0w_f	12393125	94	0.1979	0.2553	0.2227	2.0131	0.1559
M16	10	*VPS26A*	snp_rs1802295_0w_f	70931474	93	0.2995	0.4194	0.3668	1.9087	0.1671
M17	14	*H1F1A*	snp_rs2057482_0w_r	62213848	94	0.087	0.0957	0.0912	0.2376	0.6259
M18	7	*DGKB*	snp_rs2191349_0w_f	15064309	93	0.3632	0.4194	0.4769	1.3531	0.2447
M19	2	*IRS1*	snp_rs2943641_0w_r	227093745	94	0.1652	0.1809	0.1817	0.0021	0.9639
M20	4	*PPARGC1A*	snp_rs2970847_0w_f	23815924	94	0.1283	0.1489	0.1378	0.6085	0.4353
M21	5	*ARL15*	snp_rs4311394_0w_r	53300662	94	0.3283	0.3723	0.4139	0.949	0.33
M22	5	*ZBED3*	snp_rs4457053_0w_f	76424949	93	0.3747	0.4946	0.4995	0.0088	0.9253
M23	10	*TCF7L2*	snp_rs4506565_0w_f	114756041	94	0.1917	0.2447	0.2147	1.8265	0.1765
M24	3	*ADAMTS9-AS2*	snp_rs4607103_0w_r	64711904	94	0.3501	0.4149	0.4524	0.6464	0.4214
M25	11	*MTNR1B*	snp_rs4753426_0w_f	92701596	94	0.2789	0.3191	0.335	0.2103	0.6465
M26	10	*HHEX*	snp_rs5015480_0w_r	94465559	94	0.3402	0.4468	0.4346	0.0744	0.7851
M27	1	*NOTCH2*	snp_rs699780_0w_f	120455441	94	0.2705	0.3404	0.3225	0.2889	0.5909
M28	11	*LOC387761*	snp_rs7480010_0w_f	42246718	94	0.2214	0.2553	0.2535	0.0048	0.9448
M29	17	*HNF1B*	snp_rs7501939_0w_f	36101156	94	0.2943	0.4043	0.3585	1.5287	0.2163
M30	2	*THADA*	snp_rs7578597_0w_r	43732823	94	0.1041	0.117	0.1102	0.3631	0.5468
M31	6	*CDKAL1*	snp_rs7754840_0w_f	20661250	94	0.3482	0.4255	0.4491	0.2583	0.6113
M32	2	*GCKR*	snp_rs780094_0w_r	27741237	94	0.3709	0.5319	0.4919	0.6237	0.4297
M33	10	*TCF7L2*	snp_rs7903146_0w_f	114758349	94	0.1853	0.234	0.2067	1.651	0.1988
M34	12	*TSPAN8*	snp_rs7961581_0w_f	71663102	94	0.2214	0.234	0.2535	0.5542	0.4566
M35	15	*PRC1*	snp_rs8042680_0w_r	91521337	93	0.2846	0.3333	0.3437	0.0843	0.7715
M36	4	*PPARGC1A*	snp_rs8192678_0w_f	23815662	94	0.2868	0.4043	0.347	2.5603	0.1096
M37	1	*HSD11B1*	snp_rs846910_0w_f	209875254	94	0.2978	0.3936	0.3641	0.6162	0.4325
M38	7	*KLF14*	snp_rs972283_0w_r	130466854	94	0.3356	0.3191	0.4267	5.9689	0.0146
M39	16	*FT0*	snp_rs9939609_0w_r	53820527	94	0.1853	0.1915	0.2067	0.5062	0.4768

All variants are biallelic; Chr, chromosome; SNP-Array ID, single nucleotide polymorphism identfiler in the array; PP, physical position using the reference genome HG19; N, sample size; PIC, Polymorphism information content; The HWE test, Hardy-Weinberg equilibrium test with 1° of freedom; PR > ChiSq, associated p-value.

## 2 Experimental design, subjects, and methods

### 2.1 Subjects

Healthy Mexican-Maya individuals (45) and diagnosed with T2D (47) were recruited according to the following characteristics: a) having a native last name, self-classified as Maya, and having lived at the site of sampling for at least three consecutive generations. The total number of participants were 92 individuals proceeding from primary centres located in the urban and rural areas from Yucatan. This study was conducted according to the Declaration of Helsinki concerning research in humans.

### 2.2 Phenotyping

Anthropometric measurements were performed by clinical staff with a measuring tape to the nearest centimetre and weighed before breakfast under specific protocols as appropriate. Fasting plasma glucose was measured by enzymatic methods.

### 2.3 Genotyping

All participants were genotyped with the Affymetrix Axiom Genome-wide LAT1 array [World Array 4, Affymetrix, Santa Clara, CA, USA; Hoffmann et al., 2011], including 817,823 SNPs. This array was designed with a focus on the study of Latin-American populations [[10], [11], [12]].

### 2.4 Sample and SNP quality control measures

Call rates lower than 97% were eliminated from the analysis. Individuals related up to the 3rd degree were removed and duplicated or considered discordant participants according to sex markers. A significant lack of Hardy Weinberg Equilibrium was fixed at p < 10-5. Rare SNPs with a minor allele frequency <0.010 were eliminated. At the end of the filtering process, 757,439 SNPs remained for further studies.

### 2.5 Population structure analysis

To identify the population structure, 10,000 markers from the literature were selected, and after the QC process, we obtained 1289 markers by including European, African, and Native American populations. To define the number of clusters grouped in the Maya population, a five-fold cross-validation analysis was performed.

### 2.6 Statistical genetic analyses

Genotype, allelic, and Armitage-trend association models for association analysis were included using the SAS software in the CASE-CONTROL procedure (SAS/Genetics (TM) 9.4, SAS Institute, Cary, NC, USA). To identify positive association, p < 2.200 × 10^−8^ was fixed, and p < 0.001 for the replication analysis of 39 SNPs previously associated with T2D. Power analysis was performed using the Quanto software (Quanto 1.2.4, California, USA). Because the Maya population of this study is small and apparently isolated the endogamy grade was previously calculated as 1-He/Ho, where He and Ho are the expected and observed heterozygosity respectively [1]. Endogamy was null or very low since the inbreeding parameter were between −0.25 and 0.25. Consequently, the adjustment for the relationship among relatives was not needed.
